# Plant‐mediated effects of soil phosphorus on the root‐associated fungal microbiota in *Arabidopsis thaliana*

**DOI:** 10.1111/nph.15538

**Published:** 2018-11-01

**Authors:** Izabela Fabiańska, Nina Gerlach, Juliana Almario, Marcel Bucher

**Affiliations:** ^1^ Botanical Institute Cologne Biocenter University of Cologne Cologne 50931 Germany; ^2^ Cluster of Excellence on Plant Sciences (CEPLAS) University of Cologne Cologne 50931 Germany; ^3^ Present address: Center for Plant Molecular Biology University of Tübingen Tübingen 72074 Germany

**Keywords:** Brassicaceae, fungal microbiome, Helotiales, Olpidiales, phosphate starvation response (PSR), phosphorus (P), soil fertilization

## Abstract

- Plants respond to phosphorus (P) limitation through an array of morphological, physiological and metabolic changes which are part of the phosphate (Pi) starvation response (PSR). This response influences the establishment of the arbuscular mycorrhizal (AM) symbiosis in most land plants. It is, however, unknown to what extent available P and the PSR redefine plant interactions with the fungal microbiota in soil.
- Using amplicon sequencing of the fungal taxonomic marker ITS2, we examined the changes in root‐associated fungal communities in the AM nonhost species *Arabidopsis thaliana* in response to soil amendment with P and to genetic perturbations in the plant PSR.
- We observed robust shifts in root‐associated fungal communities of P‐replete plants in comparison with their P‐deprived counterparts, while bulk soil communities remained unaltered. Moreover, plants carrying mutations in the phosphate signaling network genes, *phr1*,* phl1* and *pho2*, exhibited similarly altered root fungal communities characterized by the depletion of the chytridiomycete taxon *Olpidium brassicae* specifically under P‐replete conditions.
- This study highlights the nutritional status and the underlying nutrient signaling network of an AM nonhost plant as previously unrecognized factors influencing the assembly of the plant fungal microbiota in response to P in nonsterile soil.

## Introduction

Phosphorus (P) is an essential plant nutrient, and the plant‐available form of P, that is inorganic P (Pi; orthophosphate) in the soil solution, is usually scarce in terrestrial ecosystems without fertilization, which limits plant growth (Schachtman *et al*., 1998). Plant performance and, ultimately, plant fitness strongly depend on the efficiency of the root system in scavenging Pi from the heterogeneous soil environment exhibiting spatial variability in plant‐available P (Roger *et al*., 2014; Werner *et al*., 2017). To mitigate P scarcity, plants express an array of morphological, physiological and metabolic changes, known as the phosphate starvation response (PSR) (Plaxton & Tran, 2011). Collectively, these changes enhance plant P acquisition and use efficiency, and reprioritize P allocation in the whole plant (Nussaume *et al*., 2011; Plaxton & Tran, 2011). The PSR is controlled systemically by the plant shoot P status and by the local Pi concentration, and includes, among others, increased production of lateral roots (Pérez‐Torres *et al*., 2008), organic acids (Shen *et al*., 2002), protons (Neumann & Römheld, 1999), acid phosphatases (Tran *et al*., 2010), nucleases (Raghothama, 1999) and Pi transporters involved in Pi uptake from the soil. These changes are associated with cellular reprogramming involving hundreds of PSR genes (Woo *et al*., 2012; Secco *et al*., 2013), mainly orchestrated by the transcription factor (TF) PHR1 and its homologs PHL1 and PHL2 (Rubio *et al*., 2001; Bustos *et al*., 2010; Sun *et al*., 2016), controlling the expression of *c*. 50% of all the PSR genes (Rubio *et al*., 2001; Bustos *et al*., 2010). Because high internal concentrations of P can cause toxicity symptoms in plants, the PSR is repressed transcriptionally by SPX1, which prevents PHR1 from binding to DNA, and posttranslationally by the ubiquitin conjugases PHO2 (Delhaize & Randall, 1995; Aung *et al*., 2006; Liu *et al*., 2012) and NLA (Park *et al*., 2014), which mediate the degradation of Pi transporters via ubiquitination. On the whole, these components of the PSR regulatory network allow plants to tailor P acquisition by the root to both shoot P demand and P availability in the soil.

Plant roots and their rhizosphere, that is the soil zone directly in contact with the roots, are colonized by a multitude of microorganisms (microbiota), comprising bacteria, fungi and other micro‐eukaryotes, thriving on plant‐derived carbon and competing with the plant and with each other for nutrients (Kuzyakov & Xu, 2013). There is increasing evidence that root–microbe interactions are modulated by nutrient availability in the soil, with an indication that plant and fungal communities are interlinked in their responses to soil fertilization, whereas bacterial communities behave rather independently of plants (Cassman *et al*., 2016). In particular, the concentration of available P in the soil regulates the establishment of the most ancient, widespread form of fungal symbiosis with plants, the arbuscular mycorrhizal (AM) symbiosis (Nagy *et al*., 2009; Breuillin *et al*., 2010). In this symbiosis, P controls the rhizosecretion of strigolactones, which affect the presymbiotic growth of AM fungi and stimulate root colonization (Akiyama *et al*., 2005) and the subsequent transfer of P to the plant host in exchange for photosynthates. However, beyond binary symbiotic interactions with single fungal species (Hiruma *et al*., 2016), our knowledge on how the PSR affects root‐associated fungi in a community context is still very limited. This trait is likely to be relevant for plant performance, as P deprivation also induces the synthesis of plant secondary metabolites with antimicrobial activity, such as glucosinolates and phenylpropanoids (including flavonoids; Pant *et al*., 2015), and modifies plant immunity, for example, through PHR1, which represses salicylic acid‐dependent responses and induces jasmonic acid biosynthesis genes (Castrillo *et al*., 2017), improving resistance to insect herbivory (Khan *et al*., 2016). Collectively, these observations suggest that P deprivation and the activation of the PSR determine structural shifts in the plant microbiota. Inspection of the bacterial microbiota of PSR‐compromised *Arabidopsis thaliana* in a high‐P soil indeed revealed altered root bacterial assemblages compared with wild‐type (WT) controls (Castrillo *et al*., 2017), but, beyond this observation, it is still largely unknown how naturally occurring changes in the plant PSR (triggered by changes in soil P availability) impact the interactions of plants with root‐associated bacterial and fungal consortia.

Descriptive studies of fungal communities colonizing the roots and rhizosphere of different plant species have demonstrated that these communities are not random associations of microbes, but partially organized microbial systems. Because most plant microbiota studies have focused on bacterial communities, we have little information on the cues (drivers) shaping fungal counterparts. All available studies have shown that the structure of the root‐associated fungal microbiome is strongly dependent on the soil in which the plant grows (Shakya *et al*., 2013; Coleman‐Derr *et al*., 2016; Almario *et al*., 2017). This ‘soil effect’ usually accounts for the overlapping effects of different soil characteristics associated with the soil's geographical origin and with edaphic factors, such as mineral nutrient content and, more specifically, soil P content (Almario *et al*., 2017; Robbins *et al*., 2018; Yu *et al*., 2018). Although such studies provide evidence that plants growing in soils which have been subjected to contrasting long‐term P fertilization regimes assemble different fungal microbiomes, the assembly rules and plant‐mediated effects of P remain enigmatic. Soil P is an important driver of the distribution of fungi in soil (Tedersoo *et al*., 2014; Cassman *et al*., 2016; He *et al*., 2016), and soil P effects can be amplified by plant‐mediated effects linked with the plant PSR. We thus hypothesized that soil P and the plant PSR are two pivotal factors interdependently controlling the root‐associated fungal microbiota.

In this study, we sought to determine how soil P and the plant's response to P availability collectively shape the root‐associated fungal microbiome in the AM nonhost species *A. thaliana*. Using amplicon sequencing of the fungal taxonomic marker ITS2, we explored how root‐associated fungal communities respond to short‐term soil amendment with P, and observed large differences in root and rhizosphere fungal assemblages relative to P availability. These differences were highly correlated with changes in the plant PSR. Fungal communities associated with the roots of P‐replete plants exhibited differences in diversity and abundance of particular orders. A root chytridiomycete fungus *Olpidium brassicae*, which is described as a soil‐borne obligate parasite that infects plant roots, was enriched in P‐replete plants. Network analysis further suggested an invasion of the root fungal microbiome by *O. brassicae* on P fertilization. The direct implication of PSR network genes in the structuring of root‐associated fungal communities was demonstrated with the *A. thaliana* mutants *phr1 phl1* and *pho2*, which accommodated altered fungal consortia in their roots in response to P. Collectively, these results indicate that soil P and the plant PSR signaling network jointly shape the root‐associated fungal microbiota in *A. thaliana*.

## Materials and Methods

### Plant growth conditions

The soil with low P content (0.6 mg kg^−1^) was collected in September 2014 from an experimental field at the Agroscope Research Station (Reckenholz, Zurich, Switzerland; Supporting Information Table S1; Gallet *et al*., 2003). The soil was dried at ambient temperature, sieved at 5 mm and amended with 0, 1, 20 and 50 mM P using two P sources: potassium phosphate buffer (K_2_HPO_4_/KH_2_PO_4_ buffer, ‘P_K’ treatments) or sodium phosphate buffer (Na_2_HPO_4_/NaH_2_PO_4_ buffer, ‘P_Na’ treatments). Two hundred milliliters of phosphate buffer were added to 1 kg of soil. ‘0 mM P’ treatments received 200 ml of water (‘0 mM P_water’ treatment), 1.66 mM KCl solution (‘0 mM P_K’ treatment) or 1.66 mM NaCl solution (‘0 mM P_Na’ treatment). The compositions of the amendment solutions are given in Table S2. The pH of all the solutions was adjusted to 7.5 to avoid modification of the soil pH (Fig. S1). After amendment, the soil was thoroughly mixed and left overnight before potting 350 g into 350‐ml pots.

Before planting in soil, *Arabidopsis thaliana* (Col‐0) seedlings were pregrown under sterile conditions for 2 wk. Seeds were surface sterilized in a 20% (v/v) household bleach solution for 10 min, washed four times with water and stratified at 4°C in the dark for 2 d. Seeds were then placed in Weck glass jars filled with 250 g of sterilized sand fertilized with 0.5 × Hoagland solution containing 500 μM P. Plants were grown under short‐day conditions (16 h 22°C : 8 h 18°C, light : dark, relative humidity of 70%) in a walk‐in phytochamber (Johnson Controls, Cologne, Germany). Two‐week‐old seedlings were transferred to soil pots (three seedlings per pot) and plants were grown for 8 wk under the same conditions as described above. The pots were watered every other day to maintain soil moisture at 70% of its retention capacity and were randomized every 2 d.

The experiment of soil amendment with different concentrations of P was performed once with three bulk soil pots and six planted pots per treatment, each planted pot containing three plants. Shoot samples were used to assess PSR‐related alterations in the shoot, whereas root and rhizosphere samples were used for fungal microbiome analysis, as described in Methods S1. Four to six additional pots per treatment were included to validate our experimental system by the assessment of the PSR in shoot and root by measurement of *AT4* (shoot) or *PHT1;8* (root) transcript levels and shoot P content by inductively coupled plasma‐mass spectrometry (ICP‐MS). The soil content of plant‐available P (Bray's No. 1 method; Bray & Kurtz, 1945) and soil pH (with 0.01 M CaCl_2_) were recorded in the different soil amendment treatments (Fig. S1).

The experiment with *A. thaliana* mutants was performed twice. *Arabidopsis thaliana* WT (Col‐0) and mutant lines *pho2* (Delhaize & Randall, 1995), *phr1* (SALK_067629C) and *phr1 phl1* (Bustos *et al*., 2010), reported to be affected in their PSR, were grown in soil at high‐P (50 mM P_K amended soil) and low‐P (nonamended soil, ‘0 mM P_water’) conditions as described above. Root, rhizosphere and shoot samples were collected as described in Methods S1.

### ITS2 amplicon sequencing and data analysis

The ITS2 amplicons were generated using the primers ITS9 and ITS4 as described previously (Almario *et al*., 2017; Methods S1; Table S3) and sequenced at the Cologne Center for Genomics. The ITS2 primers were selected based on the pilot test described in Methods S1 (Figs S2, S3). The fastq reads were processed in mothur v.1.37.3 (Schloss *et al*., 2009) using a custom pipeline (Almario *et al*., 2017; see Methods S1). The raw data have been submitted to the National Center for Biotechnology Information (NCBI) database under BioProjects PRJNA419296 (main project) and PRJNA419319 (ITS2 primers test), and a summary of the reads obtained is presented in Table S4. Statistical analyses were carried out in rstudio (v.3.2.1, RStudio: Integrated Development for R. RStudio, Inc., Boston, MA, USA). Bray–Curtis dissimilarities between the samples were calculated using the ‘vegdist’ function of the vegan package (Oksanen *et al*., 2016) employing log_10_(*X* + 1) transformed relative abundance operational taxonomic unit (OTU) tables. Principal coordinates analyses (PCoAs) were conducted with the ‘dudi.pco’ and ‘s.class’ functions from the ade4 package (Dray & Dufour, 2007), and nonmetric multi‐dimensional scaling (NMDS) was performed with the ‘metaMDS’ function from the vegan package. The constrained analysis of principal coordinates (CAP) was conducted with the ‘capscale’ function from the vegan package. To study the effect of different factors on the structure of fungal communities, permutational multivariate analysis of variance (PERMANOVA) on Bray–Curtis dissimilarities was conducted using the ‘adonis’ function of the vegan package (at *P *<* *0.05; 10 000 permutations). The SIMPER method from the vegan package was used to identify the OTUs contributing to group separation based on Bray–Curtis dissimilarities (Clarke, 1993). Fungal alpha diversity was estimated with Shannon's index *H* calculated in mothur. Ternary plots were generated using the ‘ggtern’ package in R (Hamilton, 2016). When indicated, means were compared using ANOVA followed by Tukey's test (*P *<* *0.05) or using the Wilcoxon rank sum test (*P *<* *0.05).

### Co‐occurrence fungal network analysis

The analysis was conducted on root, rhizosphere and bulk soil samples from P‐deprived (0 mM P_water, *n* = 9–17) and P‐replete (50 mM P_K and 50 mM P_Na, *n* = 18–25) conditions from three experiments including the ‘P amendment experiment’ and the two ‘mutant experiments’. log_10_(*X* + 1) transformed relative abundance OTU tables were used to calculate correlations between OTUs (Spearman's rank correlation coefficient ρ). Only strong correlations were considered (false discovery rate (FDR) corrected, *P *<* *0.05, ρ > |0.6|), and networks were inferred and visualized in cytoscape v.3.5.1 (Shannon *et al*., 2003). Network properties were calculated with the Network Analyzer tool in cytoscape and means were compared using the Wilcoxon rank sum test (*P *<* *0.05).

### Plant P content analysis via ICP‐MS

Plant material, 100–200 mg, was dried at 60°C for 2 d, digested in 0.5 ml of 67% nitric acid at 100°C for 30 min and diluted 1 : 10 in deionized water. Samples were analyzed using an Agilent 7700 ICP‐MS apparatus (Agilent Technologies, Tokyo, Japan) following the manufacturer's instructions.

### RNA extraction and quantitative reverse transcription‐polymerase chain reaction (qRT‐PCR)

Total RNA from plant roots and shoots was extracted and gene transcript levels were determined as described previously using the primers listed in Table S5 (Xue *et al*., 2015; Methods S1).

## Results

### Validation of an experimental system for microbiome analysis in nonsterile soils differing in P availability

The response of *A. thaliana* to P availability has mainly been investigated *in vitro*. Therefore, the first aim of our study was to develop and validate an experimental system based on a natural soil which was subject to amendments allowing the growth of P‐deprived and P‐replete *A. thaliana* plants. An agricultural low‐P soil (NK soil) was supplemented with minerals containing increasing amounts of P (Fig. S1a). Plants grown in NK soil without added P, that is growing in conditions with NaCl, KCl or water control treatments, showed low shoot biomass, accumulated low levels of P in the shoot and exhibited high transcript levels of the PSR marker genes *AT4* in the shoots and *PHT1;8* in the roots (Fig. S1b), indicating a low P status of the plant and the establishment of the plant PSR. Plants grown in soil supplied with 1 mM P, applied as sodium or potassium phosphate buffer, allocated similarly little biomass to shoots with low P concentration, and showed a small decrease in shoot *AT4* transcript abundance, whereas *PHT1;8* transcript levels in the roots remained high. By contrast, plants grown in soil amended with 20 or 50 mM P showed significantly higher biomass and P content in their shoots, which was accompanied by very low mRNA levels of *AT4* in the shoots and *PHT1;8* in the roots (Fig. S1b), indicating repression of the PSR in P‐replete plants. The results were similar irrespective of which of the two P sources was used for soil amendment, that is potassium phosphate (‘P_K’) or sodium phosphate (‘P_Na’) buffer, suggesting that the nature of the counterion to the phosphate group (K^+^ or Na^+^) had little effect on the plant PSR (Fig. S1a,b). These results demonstrated that our experimental conditions, obtained through amendment of an agricultural soil with P, triggered adaptive changes in P‐replete (20 mM and 50 mM P, inactive PSR) relative to P‐deprived (0 mM P and 1 mM P, active PSR) *A. thaliana* plants.

### Distinct fungal consortia colonize rhizosphere and root compartments in a P‐dependent manner

**Figure 1:**
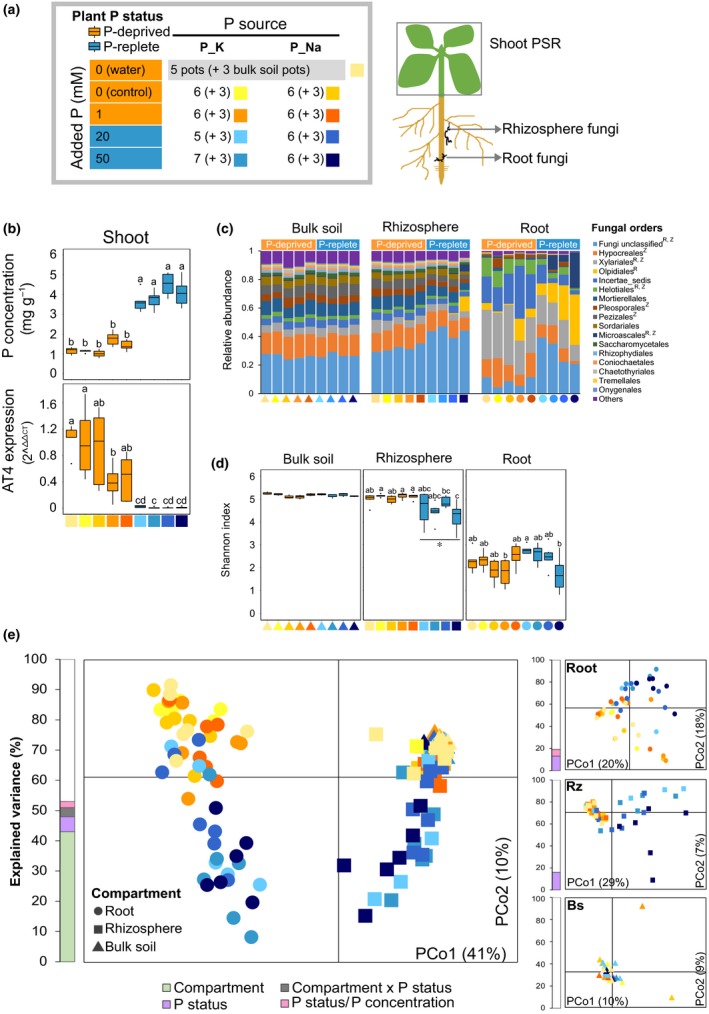
Effect of phosphorus (P) addition to soil on the plant shoot phosphate starvation response (PSR) and root‐associated fungal communities. (a) Experimental set‐up. *Arabidopsis thaliana* Col‐0 was pregrown in sterile conditions and transferred to a low‐P soil amended with increasing P concentrations (0, 1, 20 and 50 mM P in the added solution) using two P sources: potassium phosphate (‘P_K’ treatments) and sodium phosphate (‘P_Na’ treatments). Controls without added P were amended with water (‘0 mM P_water’), 1.66 mM KCl (‘0 mM P_K’) or 1.66 mM NaCl (‘0 mM P_Na’) solutions. The number of pots per treatment is indicated. Three plants were grown in each pot and the soil from unplanted pots (bulk soil) was used as a control. Bulk soil pots are indicated as ‘+ 3’ for each treatment. For sampling, the plants were pooled and shoots, roots and rhizosphere samples were taken for the different analyses. (b) Shoot P concentration and transcript levels of the shoot PSR marker gene *AT4*. Lowercase letters indicate significant differences between treatments (ANOVA, Tukey's honestly significant difference (HSD) test, *P *<* *0.05). The whiskers of the boxplots depict the dispersion of the data (1.5 × interquartile range). P‐deprived plants (orange) exhibit lower shoot P content (*t*‐test, *P *= 10^−24^) and higher *AT4* transcript levels (*t*‐test, *P *= 10^−18^) than P‐replete plants (blue). (c) Relative abundance of main fungal orders. Fungal orders significantly enriched in P‐deprived or P‐replete plants are indicated with superscripts (R for roots, Z for rhizosphere, Wilcoxon test, *P *<* *0.05). There was no difference in the abundance of fungal orders in bulk soil samples. (d) Fungal alpha diversity estimated by Shannon's diversity index. Lowercase letters indicate significant differences between treatments within compartments (ANOVA, Tukey's HSD test, *P *<* *0.05). Significant differences between P‐deprived and P‐replete plants are indicated (Wilcoxon test): *, *P *= 10^−5^. The whiskers of the boxplots depict the dispersion of the data (1.5 × interquartile range). (e) Principal coordinates analysis (PCoA) based on Bray–Curtis dissimilarities between fungal communities in root, rhizosphere (Rz) and bulk soil (Bs) compartments. Histograms placed next to the PCoA plots indicate the percentage of variability explained by each factor based on PERMANOVA (Supporting Information Table S2).

In order to study the effect of soil amendment with P on the root‐associated fungal microbiota, an experiment with the above‐described design was subsequently conducted. The harvested plant shoots were used for the analysis of the shoot PSR and the corresponding roots were processed for fungal microbiome analysis in the root (endosphere) and the rhizosphere. Unplanted pots were used for bulk soil analysis (Fig. 1a). As before, plants grown at 0 mM and 1 mM P were P‐deprived, whereas plants grown at 20 mM and 50 mM P were P‐replete (Fig. 1b).

We then assessed the responses of the fungal microbiota to changing P availability in the different microhabitats. To this end, fungal communities in the three compartments (root, rhizosphere and bulk soil) were compared considering four P concentrations (0, 1, 20 and 50 mM P) and two P sources (P_K and P_Na) as confounding factors (Table S6). Overall, the fungal communities were affected by soil P content, independent of the P source, with the compartment type as the main driver of the composition of distinctive final fungal consortia (Table S6). Fungal communities inside the root clearly differed from those in the rhizosphere and the bulk soil. Whereas *A. thaliana* rhizosphere samples exhibited a higher abundance of fungi belonging to the Hypocreales, Mortierellales, Pleosporales, Pezizales, Sordariales, Saccharomycetales, Rhizophydiales, Coniochaetales, Chaetothyriales and Tremellales, root samples were enriched in fungi belonging to the Helotiales, Microascales, Xylariales and Olpidiales (Wilcoxon test, *P *<* *0.05; Fig. 1c). Although 31 fungal OTUs from the mycorrhizal phylum Glomeromycota were detected in the soil, they showed extremely low relative abundance in *A. thaliana* roots (<0.004%) and no enrichment in root samples in comparison with the soil (0.031%) or the rhizosphere (0.008%) (Wilcoxon test, *P *>* *0.05), further sustaining the mycorrhizal nonhost status of this plant. At the OTU level, the compartment type was also the main driver of the fungal alpha diversity related to the number of taxa per sample (Shannon's *H*, ANOVA, 88% of variance, *P *=* *2 × 10^−16^). Fungal diversity decreased from the bulk soil compartment to the rhizosphere compartment and was lowest in the root (Tukey's test, *P *<* *0.05; Fig. 1d). The compartment type also strongly shaped the fungal community structure, that is the taxa present and their relative abundances (PERMANOVA on Bray–Curtis dissimilarities, 42% of variance, *P *= 10^−4^), which could also be inferred from the PCoA on Bray–Curtis dissimilarities, where bulk soil and rhizosphere samples grouped together and were separated from root samples along the first PCo (41% of variance; Fig. 1e). Similar patterns were discernible with NMDS (Fig. S4). These results showed that the root fungal community is largely determined by the compartment type and that *A. thaliana* roots are preferentially colonized by certain fungal orders.

In addition to the strong compartment differences, the amount of P added to the soil (P concentration) was the second largest factor shaping fungal communities (Fig. 1; Table S6). Although the P concentration had no significant overall effect on fungal alpha diversity (ANOVA, *P *=* *0.09), it negatively affected fungal diversity in the root‐associated compartments, with rhizosphere communities (ANOVA, 35% of variance, *P *= 10^−4^) seemingly more affected relative to root communities (ANOVA, 16% of variance, *P *=* *0.02), whereas bulk soil communities were largely unaffected (ANOVA, *P *=* *0.9; Fig. 1d; Table S6). P concentration had an overall significant effect on fungal community structure (PERMANOVA, 7% of variance *P *= 10^−4^), which was observable in the PCoA (Fig. 1e) and NMDS (Fig. S4). This effect again differed between microhabitats: it was stronger in the root and rhizosphere compartments (PERMANOVA, 19% and 20% of variance, respectively, *P *= 10^−4^) than in the bulk soil, where it was nonsignificant (PERMANOVA, *P *=* *0.8). Overall, similar results were observed with the two P sources (P_K and P_Na; Table S6), except in the case of the ‘50 mM P’ treatments (‘50 mM P_K’ and ‘50 mM P_Na’), which showed divergent fungal diversity levels in the rhizosphere compartment (Wilcoxon test, *P *=* *0.02) (Fig. 1d). Collectively, microbiome variability analysis showed that the microhabitat type and P availability in soil are key factors shaping root fungal communities. Remarkably, the observation that both root and rhizosphere fungal communities responded to short‐term P amendment, whereas bulk soil communities remained unaffected, alludes to a plant‐mediated effect of P.

### Changes in root‐associated fungal communities correlate with changes in the plant PSR

In the next step, we compared root‐associated fungal communities of P‐deprived and P‐replete plants (Table S7). Fungal communities which had established inside the roots of P‐deprived and P‐replete host plants could be clearly distinguished at the fungal order taxonomic level. In comparison with P‐deprived plants, the roots of P‐replete hosts were depleted in Helotiales and Xylariales fungi, but enriched in Microascales and Olpidiales fungi (Wilcoxon test, *P *<* *0.01). The same differences were observed in rhizosphere samples, except in the case of Olpidiales (*P *=* *0.3) (Fig. 1c). At the OTU level, treatments differing in the plant P status resulted in similarly diverse fungal communities (ANOVA, *P = *0.116), but this varied within compartments. In the rhizosphere, fungal diversity was influenced by the plant P status, as fungal communities associated with P‐replete plants (20 and 50 mM P treatments) exhibited lower diversity (Fig. 1d, Wilcoxon test, *P *= 10^−5^) in comparison with their P‐deprived counterparts (0 and 1 mM P), whereas fungal diversity in root samples remained stable (ANOVA, *P *=* *0.09). Still, the fungal community structure differed between P‐deprived and P‐replete plants (PERMANOVA, 5% of variance, *P *= 10^−4^) in both the root (PERMANOVA, 13% of variance, *P *= 10^−4^) and rhizosphere (16% of variance, *P *= 10^−4^), which could be observed in the PCoA (Fig. 1e). Nevertheless, a significant effect of the P concentration was detected within P‐deprived (0 and 1 mM P treatments) and P‐replete (20 and 50 mM P treatments) plants (PERMANOVA, 2% of variance, *P *=* *0.02) (depicted by ‘P status/P concentration’ in Fig. 1e; Table S7), indicating that the plant P status does not explain all the variation in fungal communities associated with soil amendment with P. In summary, our results showed that changes in root and rhizosphere fungal communities correlated with concurrent changes in the plant PSR, suggesting that P‐deprived and P‐replete plants selected different fungal taxa in their roots.

### Fungal taxa respond to changes in the plant PSR

**Figure 2:**
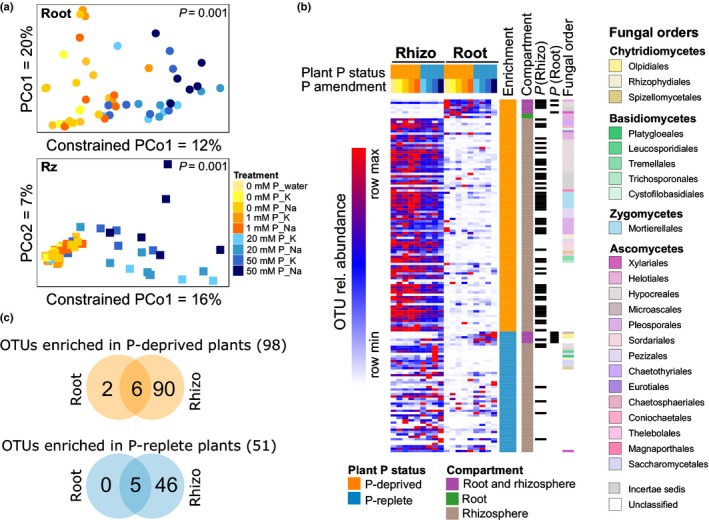
Differences between root‐associated fungal communities of phosphorus (P)‐deprived (orange) and P‐replete (blue) *Arabidopsis thaliana* plants. (a) Constrained analysis of principal coordinates (CAP), with ‘plant P status’ as a grouping variable, for root and rhizosphere (Rz) fungal communities. Samples are colored based on the treatments. The *P* value indicates the effect of the plant P status on the differences between fungal communities within compartments (ANOVA). (b) Operational taxonomic units (OTUs) enriched in P‐deprived or P‐replete roots. The heatmap represents the relative abundance of fungal OTUs contributing to the separation between P‐deprived and P‐replete plants identified by SIMPER analysis in root or rhizosphere (Rhizo) fungal communities; averages per treatment are shown. Colored stripes indicate whether the OTU was enriched in P‐deprived or P‐replete plants when considering root and/or rhizosphere samples, and provide the significance (*P*) for each of the comparisons (Wilcoxon test, *P *<* *0.05, false discovery rate (FDR) corrected). (c) Venn diagram presenting the number of fungal OTUs enriched in P‐deprived or P‐replete plants in root and rhizosphere compartments.

We further verified fungal community differences associated with P‐deprived (0 and 1 mM P) and P‐replete (20 and 50 mM P) plants by conducting CAP, which, as expected from the PERMANOVA results, showed a clear separation between the two groups for root and rhizosphere samples (Fig. 2a), but not for bulk soil samples (Fig. S5). With the aim to identify the OTUs primarily responsible for these differences (P‐responsive OTUs), we implemented a similarity percentage analysis (SIMPER; Clarke, 1993). This analysis revealed that 149 (27%) of 550 OTUs detected, on average, in *A. thaliana* roots and/or in the rhizosphere differed in their relative abundance with respect to P conditions (Fig. 2b; Table S8). Although some of these OTUs showed similar enrichment patterns in bulk soil samples, none were statistically significant (Wilcoxon test, *P *>* *0.05; Table S8), supporting the view that root and rhizosphere changes driven by P addition do not derive from changes in bulk soil fungal communities. These 149 P‐responsive OTUs accounted, on average, for 84% of the fungal reads (based on their cumulative relative abundances), suggesting that fungi which responded to the plant P status are dominant within the overall community. Among these P‐responsive OTUs, 136 (91%) contributed to differences between P‐deprived vs P‐replete plants in the rhizosphere, two (1.3%) in the root and 11 (7%) in both compartments (Fig. 2b). Furthermore, 98 OTUs (66%) were enriched in P‐deprived plants (two in the root, 90 in the rhizosphere and six in both compartments), and 51 OTUs (34%) were enriched in P‐replete plants (none in the root, 46 in the rhizosphere and five in both compartments) (Fig. 2c). These results suggested that P‐responsive fungi preferentially accumulated in the root and/or rhizosphere of P‐deprived host plants, which is in agreement with the hypothesis of a reduced activation of plant immunity during P deprivation stress (Castrillo *et al*., 2017).

**Figure 3:**
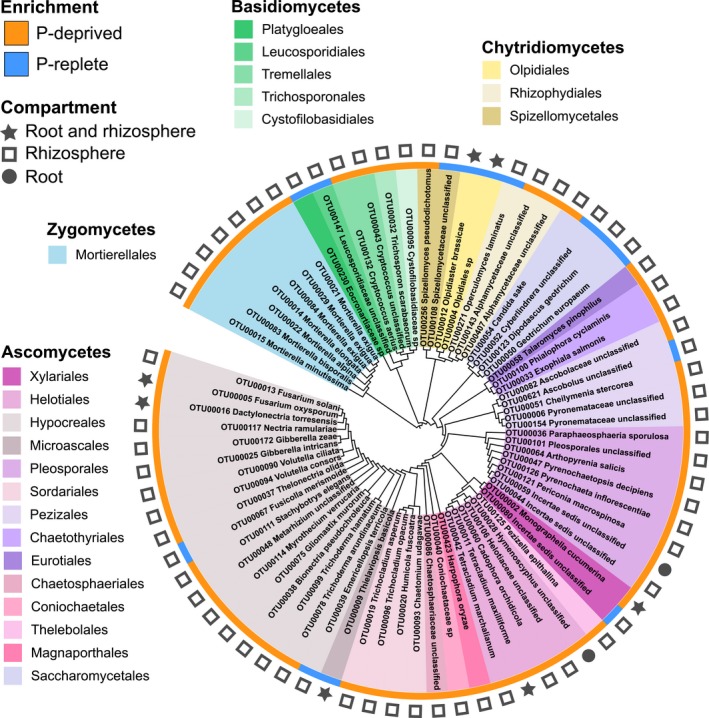
Operational taxonomic units (OTUs) associated with phosphorus (P)‐deprived or P‐replete *Arabidopsis thaliana* plants in the root and rhizosphere. Dendrogram depicting the phylogeny of fungal OTUs based on their taxonomic classification (Gower distance calculation). The colored stripe indicates whether the OTU was more abundant in P‐deprived or P‐replete plants, whereas the symbols represent the compartments: circles, root; squares, rhizosphere; stars, root and rhizosphere. Fungal OTUs are colored by their order.

The examination of the taxonomic identity of P‐responsive OTUs revealed that the above‐described shifts were caused by phylogenetically diverse OTUs belonging to members of diverse taxonomic groups, including unclassified fungi (42 OTUs), zygomycetes (7), basidiomycetes (19), chytridiomycetes (8) and mainly ascomycetes (73) (Figs 2b, S6, S7). When considering only the OTUs classified at least at the order level (Fig. 3), the largest community shifts in the rhizosphere were explained by the enrichment of OTUs belonging to Hypocreales (OTU00005, OTU00011, OTU00013), Mortierellales (OTU00014, OTU00015), Xylariales (OTU00002) and Pezizales (OTU00006) in P‐deprived plants, whereas, in the root, community changes were mostly associated with the enrichment of Hypocreales (OTU00005, OTU00016), Xylariales (OTU00002) and Helotiales (OTU00011) in P‐deprived plants and Olpidiales (OTU00004, OTU00012) in P‐replete plants (Figs 3, S7).

Notably, two of the three OTUs enriched in the roots of P‐replete plants were classified as the chytridiomycete root pathogen *Olpidiaster brassicae* (syn. *Olpidium brassicae*, OTU00012) (Hilton *et al*., 2013; Tkacz *et al*., 2015) and as an unknown Olpidiales species (OTU00004), later also classified as *O. brassicae* based on blast analysis of the representative sequence using the NCBI database (100% similarity with ITS2 sequences from *O. brassicae* isolates). *Olpidium brassicae* OTU00004 was, besides *Monographella cucumerina* OTU00002, the second most abundant OTU in the roots of P‐replete plants, accounting, on average, for 15% of the fungal reads, corresponding to a threefold increase in comparison with P‐deprived plants (5% average relative abundance of OTU00004 in roots). Collectively, these results suggested that P repletion limited root and/or rhizosphere colonization by most P‐responsive fungi across different taxonomic groups, but facilitated root colonization by a particular, potentially pathogenic, *O. brassicae*‐like taxon.

### Soil amendment with P alters the topology of fungal networks

**Figure 4:**
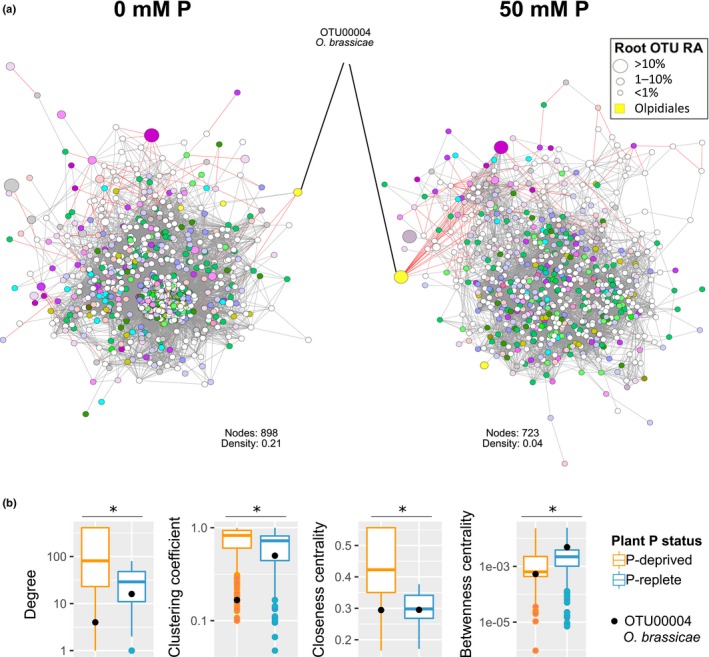
Co‐abundance networks of fungal operational taxonomic units (OTUs) in roots of phosphorus (P)‐deprived (grown in 0 mM P) and P‐replete (grown in 50 mM P_K or P_Na) *Arabidopsis thaliana* plants. (a) Networks constructed based on the correlations between fungal OTU abundances. Nodes represent OTUs and are colored by their order (as in Figs 2, 3). The size of the node indicates the relative abundance (RA) of the OTU. Edges are colored according to the Spearman correlation coefficient: red indicates negative correlation (ρ < −0.6) and gray indicates positive correlation (ρ > 0.6). (b) Differences between root fungal networks of P‐deprived (grown in 0 mM P) and P‐replete (grown in 50 mM P_K or P_Na) plants. Asterisks indicate significant differences (Wilcoxon test, *P *< 10^−16^). Black dots indicate the values for OTU00004 *Olpidium brassicae*. The whiskers of the boxplots depict the dispersion of the data (1.5 × interquartile range).

Microbe–microbe interactions are affected by environmental cues, and it was shown that continuous fertilization and other long‐term agricultural practices modify soil microbial networks (Hartmann *et al*., 2015; Ma *et al*., 2016; Morriën *et al*., 2017); however, experimental evidence for a short‐term effect of P is missing and it is unknown whether/how such changes affect fungal networks established in natural soil and within plant tissues. We computed pairwise correlations of fungal taxa in the plant root, the rhizosphere and the soil, and constructed co‐abundance networks to understand how P modified fungus–fungus interactions and how these changes related to the placement of *O. brassicae* OTU00004 in the network (Figs 4, S8, S9). To reduce the influence of the OTUs that showed little response to P conditions in the moderate treatments (1 and 20 mM P), we used only samples from contrasting P conditions (‘0 mM P water’, ‘50 mM P_K’ and ‘50 mM P_Na’) from three experiments. The comparison of co‐abundance networks of fungal OTUs in the root at low‐ and high‐P conditions indicated that P addition decreased the network's density, that is the number of connections per node, as observed by a decrease in average node degree, clustering coefficient and closeness centrality (Wilcoxon test, *P *< 10^−16^) (Fig. 4b). Except for the decrease in node degree, different trends or less pronounced differences were observed in the rhizosphere (Fig. S8) and bulk soil (Fig. S9) networks. Interestingly, the placement of *O. brassicae* OTU00004 in the root network also changed, as this node appears to be more connected (increased degree, clustering coefficient and betweenness centrality) under high‐P conditions (Fig. 4b), with an increased number of negative correlations (15 negative correlations in high‐P conditions relative to two in low‐P conditions). These results suggested that soil amendment with P not only changed the structure of the root‐associated fungal communities, but also altered the topology of the fungal networks towards reduced connectedness. In roots, these alterations overlapped with an increased abundance of *O. brassicae* and a more central placement of this taxon in the network under high‐P conditions.

### The fungal community responds to genetic perturbations in the PSR under high P

We further explored a direct involvement of the plant PSR and its genetic components in the structuring of root‐associated fungal communities using *A. thaliana* mutants defective in the central PSR regulatory pathway, that is *pho2*, with compromised phloem loading with P (a P hyperaccumulator; Delhaize & Randall, 1995; Aung *et al*., 2006), *phr1* and *phr1 phl1*, both impaired in PSR control and exhibiting reduced tissue P levels under sufficient P (Rubio *et al*., 2001; Bustos *et al*., 2010). All lines were grown in low‐P (nonamended soil, ‘0 mM P_water’) and high‐P (P‐amended soil, ‘50 mM P_K’) conditions, but *phr1 phl1* did not grow in low‐P soil. Mutant lines exhibited the expected phenotypes in terms of marker gene transcript levels and shoot P content, differing from the WT under both low‐ and high‐P conditions (Fig. S10).

Deciphering the fungal communities colonizing the root and rhizosphere of these lines in two independent experiments showed that, overall, the plant genotype (i.e. WT vs *pho*2 vs *phr1* vs *phr1 phl1*) had no effect on fungal alpha diversity (Fig. S11a), but had a small, but significant, effect on fungal community structure (PERMANOVA, 2% of variance, *P *=* *0.01; Table S9). Interestingly, the effect of the plant genotype was dependent on compartment and P condition, that is it was only significant in root samples from P‐replete soil, where the genotype explained 13% of the total variance (PERMANOVA, *P *=* *0.003; Fig. S11b; Table S10).

**Figure 5:**
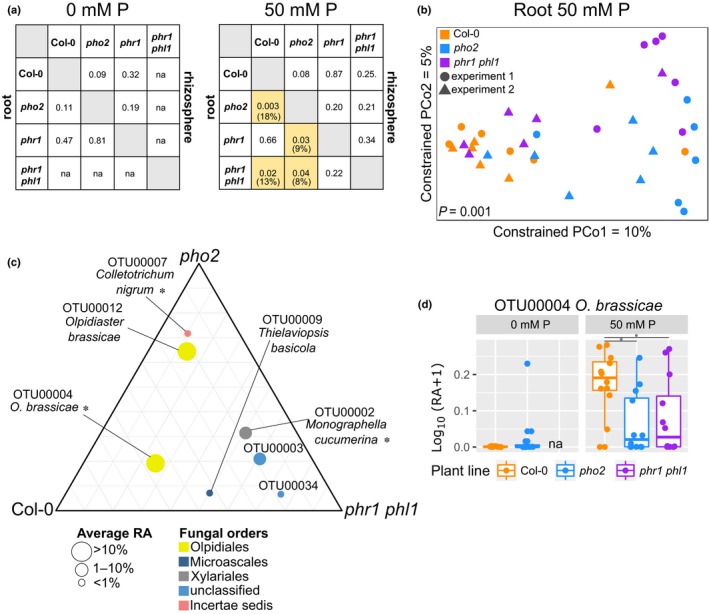
Differences in root‐associated fungal communities in *Arabidopsis thaliana* wild‐type (Col‐0) and mutant plants. Plants were pregrown in sterile conditions and transferred to low‐phosphorus (P) soil with (‘50  mM P_K’) or without (‘0 mM P_water’) P addition. (a) Pairwise comparisons of fungal communities between plant lines. *P* values (PERMANOVA) are shown and highlighted in yellow when significant (*P *<* *0.05). The percentage of the variance explained by plant line differences is given in parentheses. (b) Constrained analysis of principal coordinates (CAP) with ‘plant line’ as a grouping variable. Samples are colored based on the plant line. The *P* value is indicated for the ‘plant line’ effect on fungal community differences (ANOVA). (c) Ternary plot representing the differential abundance of selected fungal operational taxonomic units (OTUs) in root samples from three plant lines. The diameter of the circle corresponds to the mean OTU relative abundance (RA) among all plant lines. Compartments of the grid correspond to 10% increments. (d) Relative abundance of OTU00004 (Olpidiales sp.) in root samples of Col‐0, *pho2* and *phr1 phl1*. Asterisks indicate significant differences (Wilcoxon test, *P *<* *0.05). The whiskers of the boxplots depict the dispersion of the data (1.5 × interquartile range). na, no samples could be collected for *phr1 phl1* plants in 0 mM P soil. The experiment was performed twice with *n *=* *5–6 samples composed of three pooled plants.

A two‐by‐two comparison of the root and rhizosphere fungal communities associated with the different genotypes indicated that the mutation in *PHR1* failed to affect the fungal communities irrespective of the P condition, whereas mutants *pho2* and *phr1 phl1* harbored altered root fungal communities which differed significantly from the WT when grown under high‐P conditions (Fig. 5a). The differences between these three genotypes were apparent in CAP, where fungal communities in mutant and WT plants separated along the first axis and those from *phr1 phl1* and *pho2* along the second axis (Fig. 5b). The assembly of distinct fungal communities in WT and *pho2* could be explained by different expression levels of *PHO2* under high‐P conditions (Fig. S12). Thus, our results indicated that host genes which are required for plant PSR regulation were involved in the assembly of root fungal communities in P‐replete plants, whereas measurable effects were absent under P‐deprived conditions.

The mutant lines did not harbor distinct fungal communities compared with WT, in both high‐P and low‐P conditions, when examined at the fungal order level (Wilcoxon test, *P *>* *0.05; Fig. S11c). However, a SIMPER analysis allowed the identification of fungal OTUs contributing to the differences between root samples from PSR mutants and WT under high‐P conditions (Figs 5c, S13). Interestingly, most of the identified OTUs, that is OTU00003 (unclassified) and OTU00034 (unclassified), OTU00004 (*O. brassicae*), OTU00012 (*O. brassicae*) and OTU00009 (*Thielaviopsis basicola*) (Fig. 5c), were identified above as enriched in P‐replete plants (Figs 2, 3, S6). These results suggested that perturbation of the PSR specifically affected taxa which normally accumulate in WT roots under P‐replete conditions. However, disruption of key genes of the PSR did not fully explain the observed microbiome differences, as the patterns of OTU enrichment/depletion (relative to WT) in *pho2* and *phr1 phl1* differed from each other. OTU00004, classified as *O. brassicae,* an obligate Brassicaceae pathogen, was an exception and was systematically more abundant in the roots of WT (relative abundance 0.52 ± 0.28) compared with *pho2* (0.18 ± 0.23) and *phr1 phl1* (0.24 ± 0.33) (Wilcoxon test, *P *=* *0.011 and 0.046, respectively; Fig. 5d). These results imply that *A. thaliana* plants perturbed in the Pi signaling network display reduced colonization by this potential pathogenic chytridiomycete, specifically under P‐replete conditions.

## Discussion

Although the composition of the root microbiota has been shown to be strongly dependent on the soil in which the plant grows (Peiffer *et al*., 2013; Schlaeppi *et al*., 2014), the mechanistic basis underlying the control of root‐associated microbial communities by soil nutrient availability is largely unknown. Recent reports on *A. thaliana* root microbiota revealed a higher impact of long‐term soil fertilization with P on fungal communities compared with bacteria (Robbins *et al*., 2018). In this article, we significantly expanded this knowledge by studying how P availability in natural nonsterile soil and the plant's response to soil P (controlled by the PSR) jointly shape the root‐associated fungal microbiota of the AM nonhost species *A. thaliana* in the short term.

### Short‐term effects of P on root‐associated fungal communities are mediated by the plant

Soil P is an important driver of the biogeographical distribution of fungi and plants (Tedersoo *et al*., 2014). Our study revealed that short‐term P fertilization of the soil has virtually no effect on soil fungal communities, whilst causing important shifts exclusively in root‐associated (endosphere and rhizosphere) fungal communities (Fig. 1; Tables S6, S7). This contrasts with long‐term fertilization studies, which have reported shifts in fungal soil communities on long‐term amendment of the soil with P fertilizer (Leff *et al*., 2015; Robbins *et al*., 2018), and suggests that P fertilization exerts rapid effects specifically on root‐associated microbes. It is also possible that short‐term effects of P concern primarily the activity of soil fungi, rather than their presence or abundance; therefore, RNA‐ rather than DNA‐based fungal community studies are needed to account for these effects. More importantly, our results confirmed the initial hypothesis of a plant‐mediated effect of P on root‐associated fungi, with fungal communities associated with roots from P‐deprived plants clearly differing from those of their P‐replete counterparts (Figs 1, 2, 3, S4). The priming effect of the root on microbial soil communities has been well described (Huo *et al*., 2017), and we hypothesize that, similarly in our experiments, differences in root morphology and/or root exudates and rhizodeposits primed the observed shifts in fungal communities under P‐replete vs P‐deprived conditions. Plants respond to P scarcity by remodeling their root system (Williamson *et al*., 2001; Nacry *et al*., 2005), which is likely to redefine fungal niches in the root and the rhizosphere by altering the root surface area and the amount of C exuded by the roots (Holz *et al*., 2018). More specific plant responses to P scarcity include the accumulation of sugars and primary metabolites, and the production of compounds with antimicrobial activity, such as glucosinolates and flavonoids (Pant *et al*., 2015; Hiruma *et al*., 2016), which are all likely to affect plant–microbiota interactions (Badri *et al*., 2009; Bressan *et al*., 2009). Moreover, the secretion of organic acids and protons into the rhizosphere of P‐deprived plants, commonly increasing P solubility, could also impact fungal communities. In comparison with P‐replete plants, P‐deprived plants exhibited a higher fungal diversity in the rhizosphere (Fig. 1d), and the majority of the P‐responsive fungi exhibited higher relative abundance in P‐deprived plants (Fig. 2). Although relative abundance data, that is the percentage of fungal reads attributed to a given taxon, should be considered with care, as they do not reflect actual fungal loads or biological activity in plant tissues, our results suggest that the conditions in P‐deprived roots foster root colonization by more diverse fungal communities. This stands in line with the hypothesis of decreased plant immunity in P‐deprived *A. thaliana* plants exposed to bacteria (Castrillo *et al*., 2017), which could potentially facilitate fungal colonization of plant tissues under P‐limited conditions. Further research is required to specifically assess this hypothesis.

### Fungal taxa responding to alterations in the plant PSR

The fungal OTUs enriched in P‐deprived *A. thaliana* roots belonged mainly to the orders Hypocreales, Xylariales and Helotiales (Figs 1d, 2, 3; Table S8). Specifically, fungi in the Helotiales are commonly found as plant root endophytes, often in harsh environments (Zijlstra *et al*., 2005; Walker *et al*., 2011; Toju *et al*., 2016; Almario *et al*., 2017), and their proposed oligotrophic growth (Gonçalves *et al*., 2012) could explain why they are depleted on amendment of soil with P (Figs 1e, 3). Among the taxa enriched in P‐replete plants, the most characteristic alterations occurred in the order Olpidiales (Figs 1c, 2b) with a threefold enrichment of OTU00004, classified as the chytridiomycete root pathogen *Olpidium brassicae* (Figs 3, S7), in the roots. This taxon was the second most abundant OTU, accounting, on average, for 15% of the fungal reads in the root endosphere. The *Olpidium* genus includes different obligate biotrophic root pathogens and has been shown to be abundant in the Brassicaceae root microbiota in terms of DNA‐ and RNA‐based abundance (Tkacz *et al*., 2015; Gkarmiri *et al*., 2017). Our P‐replete conditions specifically promoted root colonization by this potential pathogen, favoring the inclusion of OTU00004 *O. brassicae* in the fungal community network (Fig. 4); this correlates with previous observations demonstrating the high invasion capacity of this taxon in the rhizosphere of *Brassica rapa* (Tkacz *et al*., 2015). Still, further research is needed to clarify whether this organism is indeed pathogenic to *A. thaliana* in a community context, and to understand how it escapes plant defense responses, which are expected to be stronger under P‐replete than under P‐deprived conditions.

### PSR mutants exhibit altered root fungal communities

Given the above‐mentioned findings, we hypothesized that, under P‐limited conditions, plants with a compromised response to P starvation, as described for the mutants *phr1* (Rubio *et al*., 2001) and *phr1 phl1* (Bustos *et al*., 2010), should be affected in the accommodation of root‐associated fungal communities. It was, however, impossible to comprehensively assess this hypothesis, because *phr1 phl1* plants did not survive in the low‐P (NK) soil (Fig. S10). However, when grown under P‐replete conditions, *phr1 phl1* mutant plants harbored altered root fungal communities, suggesting that these PSR regulators play an important role under P‐replete conditions in modulating plant–microbiota interactions. This is in accordance with results on bacterial communities from Castrillo *et al*. (2017), who showed that, in *A. thaliana*, PHR1 and PHL1 are involved in the regulation of plant immunity, with *phr1 phl1* mutant plants appearing to be less susceptible to tested biotrophic pathogens under P‐replete conditions. The mutation in the *PHO2* gene, which encodes a protein responsible for the degradation of Pi transporters, thus modulating Pi homeostasis under high‐P conditions (Liu *et al*., 2012), had an impact on the root fungal communities exclusively under P‐replete conditions (Fig. 5). *Pho2* mutant plants respond like WT plants to low‐P conditions and like P‐deprived plants to P‐replete conditions, where they overaccumulate P (Bari *et al*., 2006). Interestingly, in roots of both types of PSR mutants (*phr1 phl1* and *pho2*), we detected a reduction in the abundance of a potentially pathogenic Olpidiales taxon (*O. brassicae*), suggesting that, under high‐P conditions, plants with a disturbed PSR are less preferentially colonized by this fungus. Results from microbiome studies suffer from low reproducibility (Schloss, 2018). Therefore, the two studies, Castrillo *et al*. (2017) on bacterial communities and our study on fungal communities, now independently show that genetic components underlying the PSR largely affect root‐associated microbial communities, providing a framework for the robustness and generalizability of this research across the fungal and bacterial kingdoms.

Our study highlights a general control mechanism based on host genetic factors, which regulate the PSR on interaction with soil P, involved in the shaping of the fungal microbiota in plants. Further research is needed to understand the functional consequences of these interactions for plant performance and, more specifically, the importance of the root microbiota for plant growth in response to variable nutrient availability under field conditions.

## Author contributions

IF, JA and MB designed the experiments. IF and JA performed the experiments and carried out the data analysis. NG performed multi‐elemental analytics (ICP‐MS). IF, JA and MB wrote the paper.

## Supporting information

Please note: Wiley Blackwell are not responsible for the content or functionality of any Supporting Information supplied by the authors. Any queries (other than missing material) should be directed to the *New Phytologist* Central Office.

**Fig. S1** Changes in plant shoot and root phosphate starvation response (PSR) on soil amendment with phosphorus (P) (validation of the experimental system).
**Fig. S2** Comparison of primers ITS9, fITS7 and gITS7 in combination with ITS4 for fungal ITS2 amplification.
**Fig. S3** Final rarefaction curves obtained with primer set ITS4/ITS9 in bulk soil (*n *=* *39), rhizosphere (*n *=* *138) and root (*n *=* *138) samples.
**Fig. S4** Nonmetric multi‐dimensional scaling (NMDS) ordination of fungal communities in root, rhizosphere (Rz) and bulk soil (Bs) based on Bray–Curtis dissimilarities.
**Fig. S5** Constrained analysis of principal coordinates (CAP) of fungal communities in bulk soil samples.
**Fig. S6** Operational taxonomic units (OTUs) associated with phosphorus (P)‐deprived and P‐replete plants in the root and rhizosphere (including unclassified OTUs).
**Fig. S7** Relative abundance (RA) of operational taxonomic units (OTUs) contributing to differences between phosphorus (P)‐deprived and P‐replete plants based on SIMPER analysis.
**Fig. S8** Co‐abundance networks of fungal operational taxonomic units (OTUs) in rhizospheres of phosphorus (P)‐deprived (grown in 0 mM P) and P‐replete (50 mM P_K or P_Na) plants.
**Fig. S9** Co‐abundance networks of fungal operational taxonomic units (OTUs) in bulk soil under phosphorus (P)‐deprived (grown in 0 mM P) and P‐replete (50 mM P_K or P_Na) conditions.
**Fig. S10** Physiological responses of wild‐type (WT) and mutant plants grown in soil under phosphorus (P)‐deprived and P‐replete conditions.
**Fig. S11** Root‐associated fungal communities in *Arabidopsis thaliana*wild‐type (Col‐0) and mutant plants.
**Fig. S12** *PHO2* gene expression in roots.
**Fig. S13** Relative abundance (RA) of operational taxonomic units (OTUs) contributing to differences between plant lines under high‐phosphorus (P) conditions (‘50 mM P_K’) based on SIMPER analysis.
**Methods S1** Methods for root, rhizosphere and shoot sample collection; ITS2 amplicon sequencing; processing of ITS2 amplicon data; RNA extraction and quantitative reverse transcription‐polymerase chain reaction (qRT‐PCR).
**Table S1** Physicochemical characteristics of the soil used.
**Table S2** Solutions used for soil amendment.
**Table S3** Tagged primers used to prepare the ITS2 amplicon libraries.
**Table S4** Sequence analysis summary.
**Table S5** Primers used for quantitative reverse transcription‐polymerase chain reaction (qRT‐PCR).
**Table S6** Effect of different factors on the alpha diversity (ANOVA on Shannon's *H* index) and structure (PERMANOVA on Bray–Curtis dissimilarities) of fungal communities in the soil phosphorus (P) amendment experiment.
**Table S7** Effect of different factors on the alpha diversity (ANOVA on Shannon's *H* index) and structure (PERMANOVA on Bray–Curtis dissimilarities) of fungal communities in the soil phosphorus (P) amendment experiment (the plant P status was included as a factor; see Figs 1, S4, S5).
**Table S9** Effect of different factors on the alpha diversity (ANOVA on Shannon's *H* index) and structure (PERMANOVA on Bray–Curtis dissimilarities) of fungal communities in the *Arabidopsis thaliana* mutants experiment (see Figs 5, S11).
**Table S10** Within‐treatment analysis of the effect of different factors on the alpha diversity (ANOVA on Shannon's *H* index) and structure (PERMANOVA on Bray–Curtis dissimilarities) of fungal communities in the *Arabidopsis thaliana* mutants experiment (see Figs 5, S11).

**Table S8** Fungal operational taxonomic units (OTUs) identified by SIMPER analysis as enriched in phosphorus (P)‐deprived or P‐replete conditions.
