# Supplementary material for: Plant‐mediated effects of soil phosphorus on the root‐associated fungal microbiota in Arabidopsis thaliana
Source: New Phytol. 2018 Nov 1;221(4):2123–37. doi: 10.1111/nph.15538 (PMC6519159; doi:10.1111/nph.15538)
Supplement: Supplementary file 1 — Fig. S1 Changes in plant shoot and root phosphate starvation response (PSR) on soil amendment with phosphorus (P) (validation of the experimental system). Fig. S2 Comparison of primers ITS9, fITS7 and gITS7 in combination with ITS4 for fungal ITS2 amplification. Fig. S3 Final rarefaction curves obtained with primer set ITS4/ITS9 in bulk soil (n = 39), rhizosphere (n = 138) and root (n = 138) samples. Fig. S4 Nonmetric multi‐dimensional scaling (NMDS) ordination of fungal communities in root, rhizosphere (Rz) and bulk soil (Bs) based on Bray–Curtis dissimilarities. Fig. S5 Constrained analysis of principal coordinates (CAP) of fungal communities in bulk soil samples. Fig. S6 Operational taxonomic units (OTUs) associated with phosphorus (P)‐deprived and P‐replete plants in the root and rhizosphere (including unclassified OTUs). Fig. S7 Relative abundance (RA) of operational taxonomic units (OTUs) contributing to differences between phosphorus (P)‐deprived and P‐replete plants based on SIMPER analysis. Fig. S8 Co‐abundance networks of fungal operational taxonomic units (OTUs) in rhizospheres of phosphorus (P)‐deprived (grown in 0 mM P) and P‐replete (50 mM P_K or P_Na) plants. Fig. S9 Co‐abundance networks of fungal operational taxonomic units (OTUs) in bulk soil under phosphorus (P)‐deprived (grown in 0 mM P) and P‐replete (50 mM P_K or P_Na) conditions. Fig. S10 Physiological responses of wild‐type (WT) and mutant plants grown in soil under phosphorus (P)‐deprived and P‐replete conditions. Fig. S11 Root‐associated fungal communities in Arabidopsis thalianawild‐type (Col‐0) and mutant plants. Fig. S12 PHO2 gene expression in roots. Fig. S13 Relative abundance (RA) of operational taxonomic units (OTUs) contributing to differences between plant lines under high‐phosphorus (P) conditions (‘50 mM P_K’) based on SIMPER analysis. Methods S1 Methods for root, rhizosphere and shoot sample collection; ITS2 amplicon sequencing; processing of ITS2 amplicon data; RNA extraction a [file NPH-221-2123-s001.pdf]

New Phytologist **Supporting Information**

Article title: Plant-mediated effects of soil phosphorus on the root-associated fungal microbiota in *Arabidopsis thaliana*
Authors: Izabela Fabiańska, Nina Gerlach, Juliana Almario, Marcel Bucher
Article acceptance date: 19 September 2018

The following Supporting Information is available for this article:

Fig. S1 Changes in plant shoot and root PSR upon soil amendment with P (validation of the experimental system).

Fig. S2 Comparison of primers ITS9, fITS7 and gITS7 in combination with ITS4 for fungal ITS2 amplification.

Fig. S3 Final rarefaction curves obtained with primer set ITS4/ITS9 in bulk soil (n = 39), rhizosphere (*n* = 138) and root (*n* = 138) samples.

Fig. S4 NMDS ordination of fungal communities in root, rhizosphere (Rz) and bulk soil (Bs) based on Bray-Curtis dissimilarities.

Fig. S5 Constrained Analysis of Principal Coordinates (CAP) of fungal communities in bulk soil samples.

Fig. S6 OTUs associated to P-deprived or P-replete plants in the root and rhizosphere (including unclassified OTUs).

Fig. S7 Relative abundance (RA) of OTUs contributing to differences between P-deprived and P-replete plants based on SIMPER analysis (excel spreadsheet, included separately).

Fig. S8 Co-abundance networks of fungal OTUs in rhizospheres of P-deprived (grown in 0 mM P) and P-replete plants (50 mM P_K or P_Na).

Fig. S9 Co-abundance networks of fungal OTUs in bulk soil under P-deprived (grown in 0 mM P) and P-replete conditions (50 mM P_K or P_Na).

Fig. S10 Physiological responses of WT and mutant plants grown in soil under P-deprived and

P-replete conditions.

Fig. S11 Root-associated fungal communities in *A. thaliana* wild type (Col-0) and mutant plants.

Fig. S12 *PHO2* gene expression in roots.

Fig. S13 Relative abundance (RA) of OTUs contributing to differences between plant lines under high-P conditions ('50 mM P_K') based on SIMPER analysis.

Table S1 Physico-chemical characteristics of the soil used.

Table S2 Solutions used for soil amendment.

Table S3 Tagged primers used to prepare the ITS2 amplicon libraries.

Table S4 Sequence analysis summary.

Table S5 Primers used for RT qPCR.

Table S6 Effect of different factors on the alpha-diversity (ANOVA on Shannon's H index) and structure (PerMANOVA on Bray-Curtis dissimilarities) of fungal communities in the soil P amendment experiment.

Table S7 Effect of different factors on the alpha-diversity (ANOVA on Shannon's H index) and structure (PerMANOVA on Bray-Curtis dissimilarities) of fungal communities in the soil P amendment experiment (The plant P-status was included as a factor; See Figs 1, S4, S5).

Table S8 Fungal OTUs identified by SIMPER analysis as enriched in P-deprived or P-replete conditions (excel spreadsheet, included separately).

Table S9 Effect of different factors on the alpha-diversity (ANOVA on Shannon's H index) and structure (PerMANOVA on Bray-Curtis dissimilarities) of fungal communities in the *A. thaliana* mutants experiment (See Figs 5, S11).

Table S10 Within-treatment analysis of the effect of different factors on the alpha-diversity (ANOVA on Shannon's H index) and structure (PerMANOVA on Bray-Curtis dissimilarities) of

fungal communities in the *A. thaliana* mutants experiment (See Figs 5, S11).

Methods S1 Methods for root, rhizosphere and shoot sample collection; ITS2 amplicon sequencing; processing ITS2 amplicon data; RNA extraction and qRT-PCR.

Methods S1

Root, rhizosphere and shoot sample collection

Plants were dug-out from the pots and the loosely attached soil was removed by shaking the roots. The root systems of three plants per pot were pooled, placed in 15 ml Falcon tubes containing 3 ml sterile PBS buffer (130 mM NaCl, 7 mM Na_2HPO_4, 3 mM NaH_2PO_4, pH 7.0, 0.02% Silwet L-77) and shaken for 20 min at 180 rpm for washing. The soil solution obtained from this first washing was centrifuged (1,500 x g, 20 min) and the soil pellet, defined as the rhizosphere sample, was frozen in liquid nitrogen. The roots were washed three more times in PBS buffer, blot dried and frozen in liquid nitrogen. Three bulk soil samples per treatment were collected from the center of unplanted pots after removing the first 1 cm top layer of soil. The DNA of root, rhizosphere and bulk soil samples extracted using the FastDNA SPIN Kit for Soil (MP Biomedicals, Solon, USA) according to the manufacturers' instructions. Three plant shoots per pot were pooled, washed in water, blot dried and grinded in liquid nitrogen before subsampling for RNA extraction and ICP-MS.

For PSR analysis, shoots and roots from one pot were separately pooled, washed in water, blot dried and frozen in liquid nitrogen before grinding and subsampling for RNA extraction and ICP-MS.

ITS2 amplicon sequencing

In a pilot experiment a suitable primer pair for fungal ITS2 sequencing from *A. thaliana* root and rhizosphere samples was selected. The forward primers ITS9, fITS7 and gITS7 proposed by Ihrmark et al. (2012) were tested in combination with the reverse primer ITS4 (Gardes and Bruns, 1993), on three root and three rhizosphere samples and the sequencing outputs were compared. ITS2 amplicons were generated using a two-step PCR method. The first PCR step was performed with non-tagged primers in a Verity Thermal Cycler (Applied Biosystems, Carlsbad, CA, USA) in 25 µL reactions containing 20 ng of DNA template, 200 µM of each nucleotide, 2.75 mM $MgCl_2$, 0.1 U μL^{-1} of G2 Flexi DNA Polymerase (Promega, Mannheim,

Germany) and 1X G2 Flexi DNA Polymerase buffer. The primer concentrations were 1000 nM, 500 nM and 500 nM, for ITS9, fITS7 and gITS forward primer, respectively, and 300 nM for ITS4 reverse primer. Annealing temperature and cycle number were adjusted to each primer pair: 55°C and 30 cycles for ITS9/ITS4, 57°C and 26 cycles for fITS7/ITS4, 56°C and 25 cycles for gITS7/ITS4. Cycling conditions were 5 min at 95 ^{o}C, 25 to 30 cycles of 30 sec at 95 ^{o}C, 30 sec at 55 ^{o}C to 57 ^{o}C and 30 sec at 72 ^{o}C, followed by a final elongation of 7 min at 72°C. The PCR product from the first PCR step was diluted 1:5 with water and 5 µL were used as template in the second PCR step. PCR conditions were the same as in the first PCR step, except that amplification was limited to 5 cycles and tagged primers were used (Table S3; for forward primers fITS7 and gITS7 the same tag sequences were used). PCR products were checked by agarose gel electrophoresis, the four technical replicates per sample were pooled and 30 µL were used for PCR product purification using Agencourt AMPure XP PCR purification (Beckmen Coulter, Krefeld, Germany) following the manufacturer's instructions. Amplicon concentrations were measured by NanoDrop and libraries were prepared by pooling 12 tagged samples in equimolar amounts. Finally, libraries were purified with NucleoSpin Gel and PCR Clean-up kit (Macherey Nagel, Dueren, Germany) and were indexed using the MiSeq Reagent Kit v3 (Illumina, San Diego, CA, USA) before sequencing in a single MiSeq run with 3% Phix control at the Cologne Center for Genomics (University of Cologne, Cologne, Germany). For the main experiments, ITS2 amplification was conducted as described above with primers ITS9/ITS4 since they exhibited less plant ITS2 amplification and a better recovery of fungal diversity, i.e. more fungal orders were detected, in comparison to the other primers tested (Figure S2). The final libraries were divided into two MiSeq runs.

Processing ITS2 amplicon data

ITS2 amplicon sequencing data was analyzed in Mothur version 1.37.3 (Schloss et al., 2009). Forward and reverse paired end reads were assembled into contigs and contigs with less than 5 nt mismatch-free overlap between forward and reverse reads were discarded. Reads were filtered to a maximum of two mismatches with the primer sequence, a minimum length of 100 nt, a maximum of 1 ambiguous base (N) and a maximum homopolymer content of 8 nt. Reads differing by less than 3 nt were then clustered together and possible chimeras were identified and removed using UCHIME with abundant sequences as reference. Sequences were

clustered into OTUs at 97% sequence similarity using VSEARCH with the abundance greedy clustering method (agc). Each OTU was taxonomically classified with 60% confidence level after classifying each sequence using the RDP Bayesian Classifier and the UNITE fungal ITS database (Version 7.0, release 31.01.2016; Kõljalg et al., 2013). The *A. thaliana* ITS2 sequence was included in the database to better identify plant sequences. For each OTU, the most abundant sequence was picked as the representative sequence. Low abundant OTUs with less than 50 reads, OTUs unclassified at the kingdom level and plant OTUs were discarded from the final OTU table. The relative abundance of each OTU in each sample was calculated from this final OTU table. For each sample, the Shannon diversity index (H') was calculated and a rarefaction curve was generated using Mothur.

We analyzed fungal communities present in soil and in *A. thaliana* root and rhizosphere under different amendment regimes with P. We collected in total 138 root, 138 rhizosphere and 39 bulk soil samples in which the fungal communities were analyzed using the above described Illumina-based ITS2 amplicon sequencing. We obtained 6 829 927 high quality reads that were clustered using de novo OTU picking at 97% sequence similarity. After removal of low abundance OTUs with less than 50 reads, this resulted in an average of 708 fungal OTUs per sample (Table S4) with root samples hosting significantly less OTUs (on average 148 OTUs) than bulk soil and rhizosphere samples (on average 949 OTUs) (Figure S3). In sterile grown plants (before transfer into the soil) only 53 fungal reads were detected per plant at most, indicating virtually no contamination.

RNA extraction and qRT-PCR

Total RNA from plant roots and shoots was extracted using the NucleoSpin RNA Plant extraction kit (Macherey Nagel, Dueren, Germany) according to the manufacturer's instructions. Genomic DNA digestion was performed by incubating 250 ng of total RNA with 1 µl DNase (Thermo Fisher Scientific, USA) according to the manufacturer's instructions. DNA-free RNA samples were reverse transcribed into cDNA using the Thermo Scientific RevertAid H Minus Reverse Transcriptase (Applied Biosystems, Cheshire, UK). Transcript levels were determined by quantitative real-time (qRT-) PCR in an ABI 7500 thermo cycler (Applied Biosystems, Foster City, USA) using SYBR green PCR master mix (Applied Biosystems, Cheshire, UK) with three technical replicates. Relative transcript levels were calculated using the 2-ΔΔCt

method (Livak and Schmittgen, 2001), using Ubq10 transcripts as reference. The primers used are listed in Table S5.

References

Gardes M, Bruns TD. 1993. ITS primers with enhanced specificity for basidiomycetes-application to the identification of mycorrhizae and rusts. *Molecular Ecology* **2**: 113–118.

Almario J, Jeena G, Wunder J, Langen G, Zuccaro A, Coupland G, Bucher M. 2017. Root-associated fungal microbiota of nonmycorrhizal *Arabis alpina* and its contribution to plant phosphorus nutrition. *Proceedings of the National Academy of Sciences, USA* **114**: E9403–E9412.

Balcerowicz M, Ranjan A, Rupprecht L, Fiene G, Hoecker U. 2014. Auxin represses stomatal development in dark-grown seedlings via Aux/IAA proteins. *Development* **141**: 3165–3176.

Gloor GB, Hummelen R, Macklaim JM, Dickson RJ, Fernandes AD, MacPhee R, Reid G. 2010. Microbiome profiling by Illumina sequencing of combinatorial sequence-tagged PCR products. *PLOS ONE* **5**: e15406.

Ihrmark K, Bödeker ITM, Cruz-Martinez K, Friberg H, Kubartova A, Schenck J, Strid Y, Stenlid J, Brandström-Durling M, Clemmensen KE *et al.* 2012. New primers to amplify the fungal ITS2 region – evaluation by 454-sequencing of artificial and natural communities. *FEMS Microbiology Ecology* **82**: 666–677.

Kõljalg U, Nilsson RH, Abarenkov K, Tedersoo L, Taylor AFS, Bahram M, Bates ST, Bruns TD, Bengtsson-Palme J, Callaghan TM *et al.* 2013. Towards a unified paradigm for sequence-based identification of fungi. *Molecular Ecology* **22**: 5271–5277.

Nilsson L, Müller R, Nielsen TH. 2007. Increased expression of the MYB-related transcription factor, PHR1, leads to enhanced phosphate uptake in *Arabidopsis thaliana*. *Plant, Cell & Environment* **30**: 1499–1512.

Livak KJ, Schmittgen TD. 2001. Analysis of relative gene expression data using real-time quantitative PCR and the 2-Delta Delta CT Method. *Methods* **25**: 402–408.

Schloss PD, Westcott SL, Ryabin T, Hall JR, Hartmann M, Hollister EB, Lesniewski RA, Oakley BB, Parks DH, Robinson CJ *et al.* 2009. Introducing mothur: Open-source, platform-independent, community-supported software for describing and comparing microbial communities. *Applied and Environmental Microbiology* **75**: 7537–7541.

Fig. S1 Changes in plant shoot and root PSR upon soil amendment with P (validation of the experimental system). (a) Plant-available soil P and pH were measured in bulk soil before planting *Arabidopsis thaliana* seedlings (n = 3). **(b)** Shoot fresh weight (FW), shoot P concentration, *AT4* transcript levels in shoots and *PHT1.8* transcript levels in roots. Means and standard errors are shown, a-c indicate significant differences between the treatments (ANOVA, Tukey's test, $P < 0.05$). Four to six additional pots per treatment, included in the P-amendment experiment were used for this analysis. n = 4 - 6 samples composed of three pooled plants per pot.

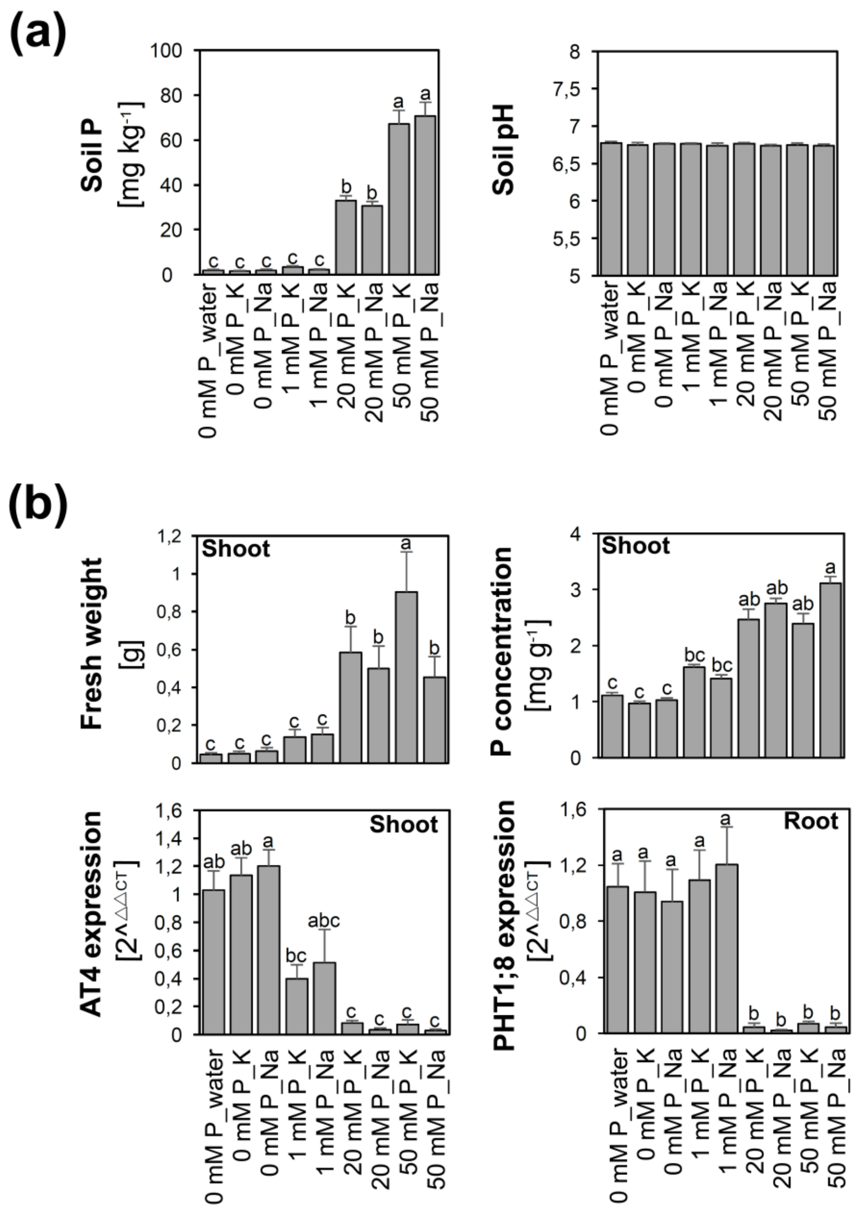

Fig. S2 Comparison of primers ITS9, fITS7 and gITS7 in combination with ITS4 for fungal ITS2 amplification. (a) Sequence similarities between the primers and their binding site on *A. thaliana* ITS2. Partially or fully mismatching bases are indicated in red. **(b)** Percentage of fungal, plant and 'unknown' (i.e. unclassified at the kingdom level) ITS2 sequences amplified by the tested primers. **(c)** Number of fungal orders recovered by the tested primers in root and rhizosphere samples. Means and standard errors are shown, a-c indicate significant differences (ANOVA followed by Tukey's test, $P < 0.05$). The experiment was performed once with $n = 3$ samples composed of three pooled plants.

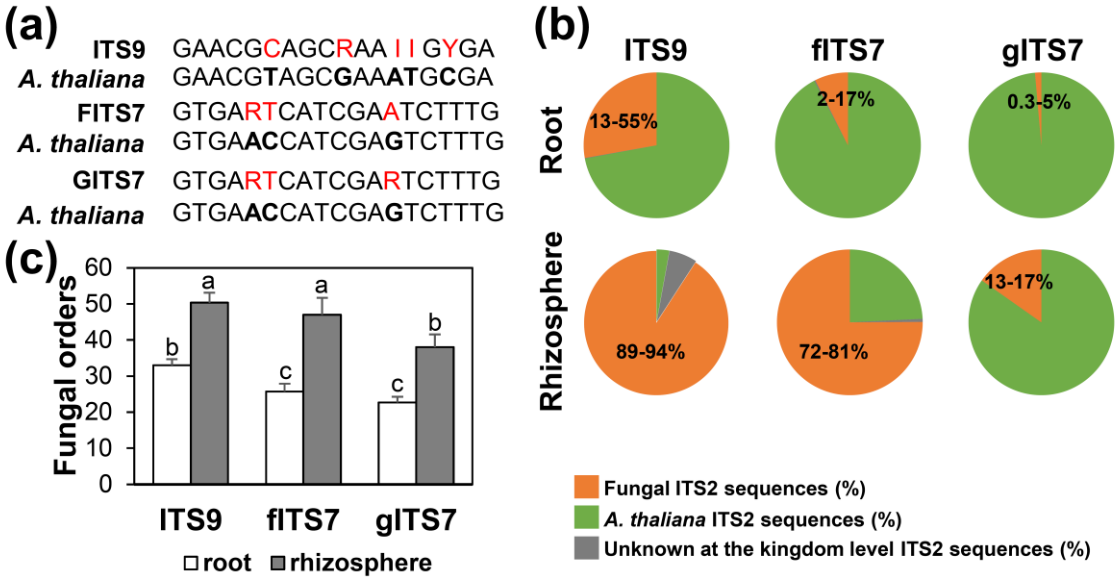

Fig. S3 Final rarefaction curves obtained with primer set ITS4/ITS9 in bulk soil (n = 39), rhizosphere (*n* = 138) and root (*n* = 138) samples.

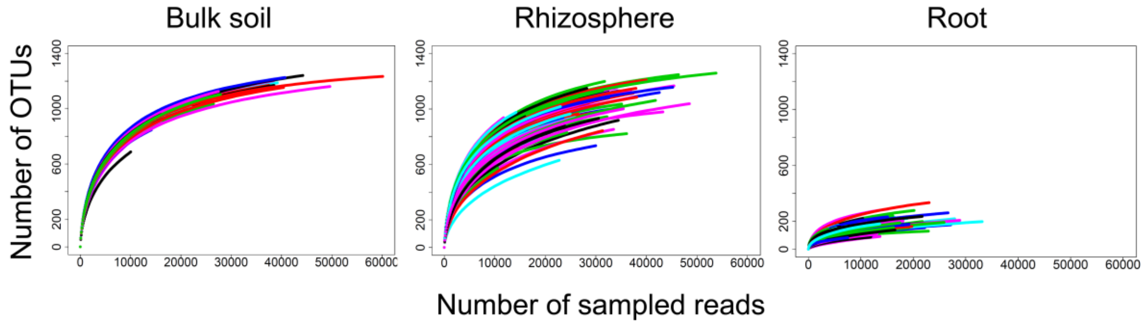

Fig. S4 NMDS ordination of fungal communities in root, rhizosphere (Rz) and bulk soil (Bs) based on Bray-Curtis dissimilarities.

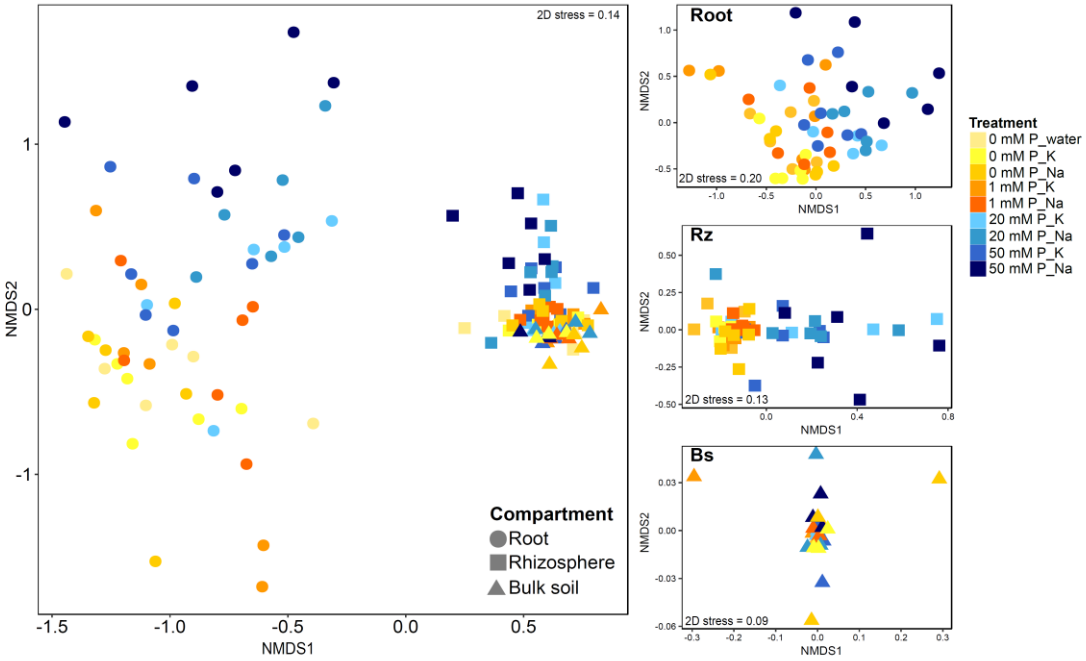

Fig. S5 Constrained Analysis of Principal Coordinates (CAP) of fungal communities in bulk soil samples. Bray- Curtis dissimilarities were used as a distance metric and soil P status (P-deprived : 0 mM and 1 mM P; P-replete : 20 mM and 50 mM P) as a grouping variable. The *P*-value indicates the significance of the effect of the soil P-status (P-deprived *vs* P-replete) on sample separation along the x axis (ANOVA).

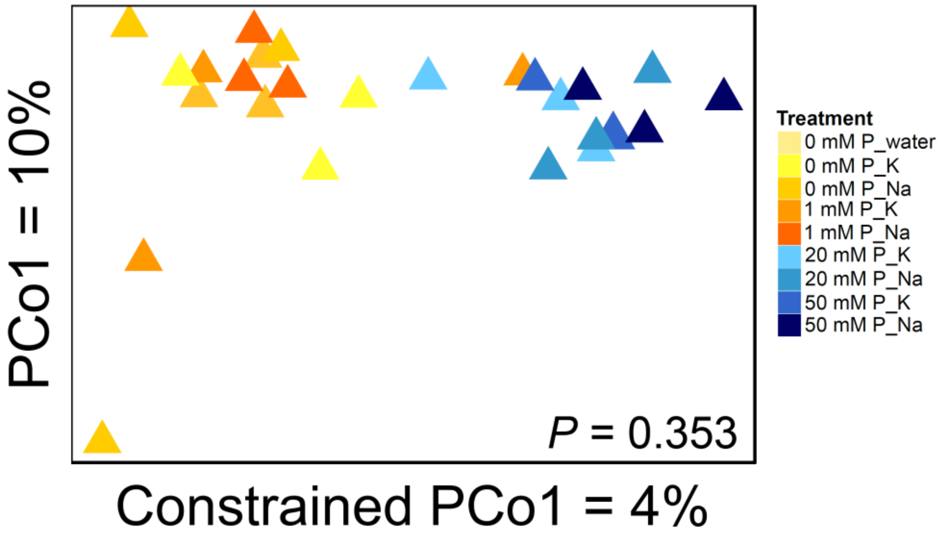

Fig. S6 OTUs associated to P-deprived or P-replete plants in the root and rhizosphere (including unclassified OTUs). Dendrogram depicting fungal OTUs phylogeny based on their taxonomic classification (Gower distance calculation). The colored stripe indicates if the OTU was more abundant in P-deprived or P-replete *A. thaliana* plants, whereas symbols represent the compartments (circle - root, square - rhizosphere, star - root and rhizosphere). Fungal OTUs are colored by their order.

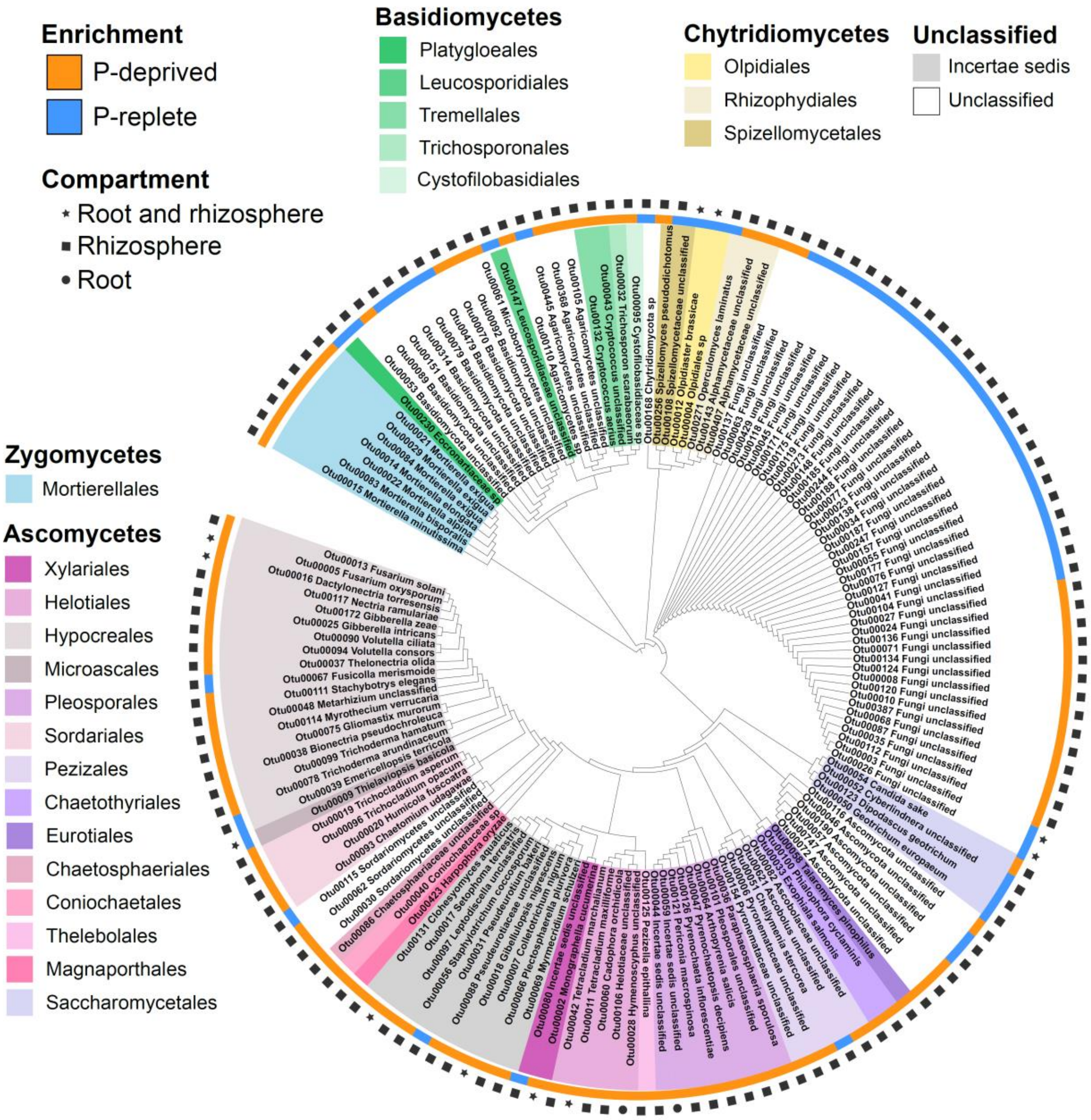

Fig. S7 Relative abundance (RA) of OTUs contributing to differences between P-deprived and P-replete plants based on SIMPER analysis. The OTUs showing a significantly different RA between P-deprived and P-replete *A. thaliana* plants in root or in rhizosphere are indicated with an asterisk (Wilcoxon $P < 0.05$, brown asterisk for rhizosphere, green for root). The whiskers of the box-plots depict the dispersion of the data (1.5 × interquartile range).

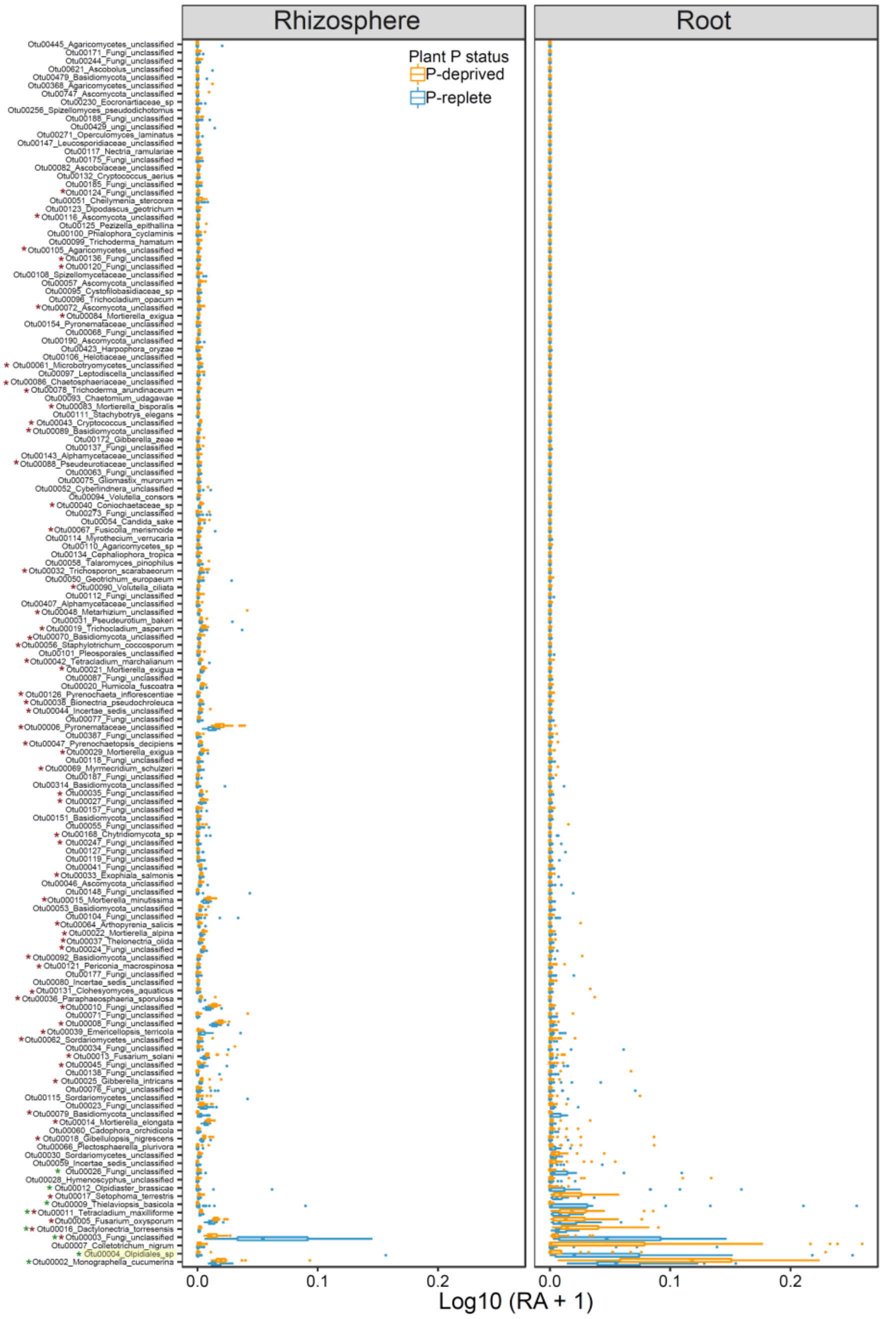

Fig. S8 Co-abundance networks of fungal OTUs in the rhizosphere of P-deprived (grown in 0 mM P) and P-replete plants (50 mM P_K or P_Na). (a) Networks constructed based on the correlations between fungal OTUs abundances. Nodes represent OTUs and are colored by their order (as in Figs. 2, 3). The size of the node indicates the relative abundance (RA) of the OTU. Edges are colored accordingly to the Spearman correlation coefficient: red indicates negative correlation ($\rho < -0.6$) and grey indicates positive correlation ($\rho > 0.6$). **(b)** Differences between rhizosphere fungal networks of P-deprived (grown in 0 mM P) and P-replete plants (50 mM P_K or P_Na). Asterisks indicate significant differences (Wilcoxon test $P < 10^{-16}$). Black dots indicate the values for Otu00004 *O. brassicae*. The whiskers of the box-plots depict the dispersion of the data (1.5 × interquartile range).

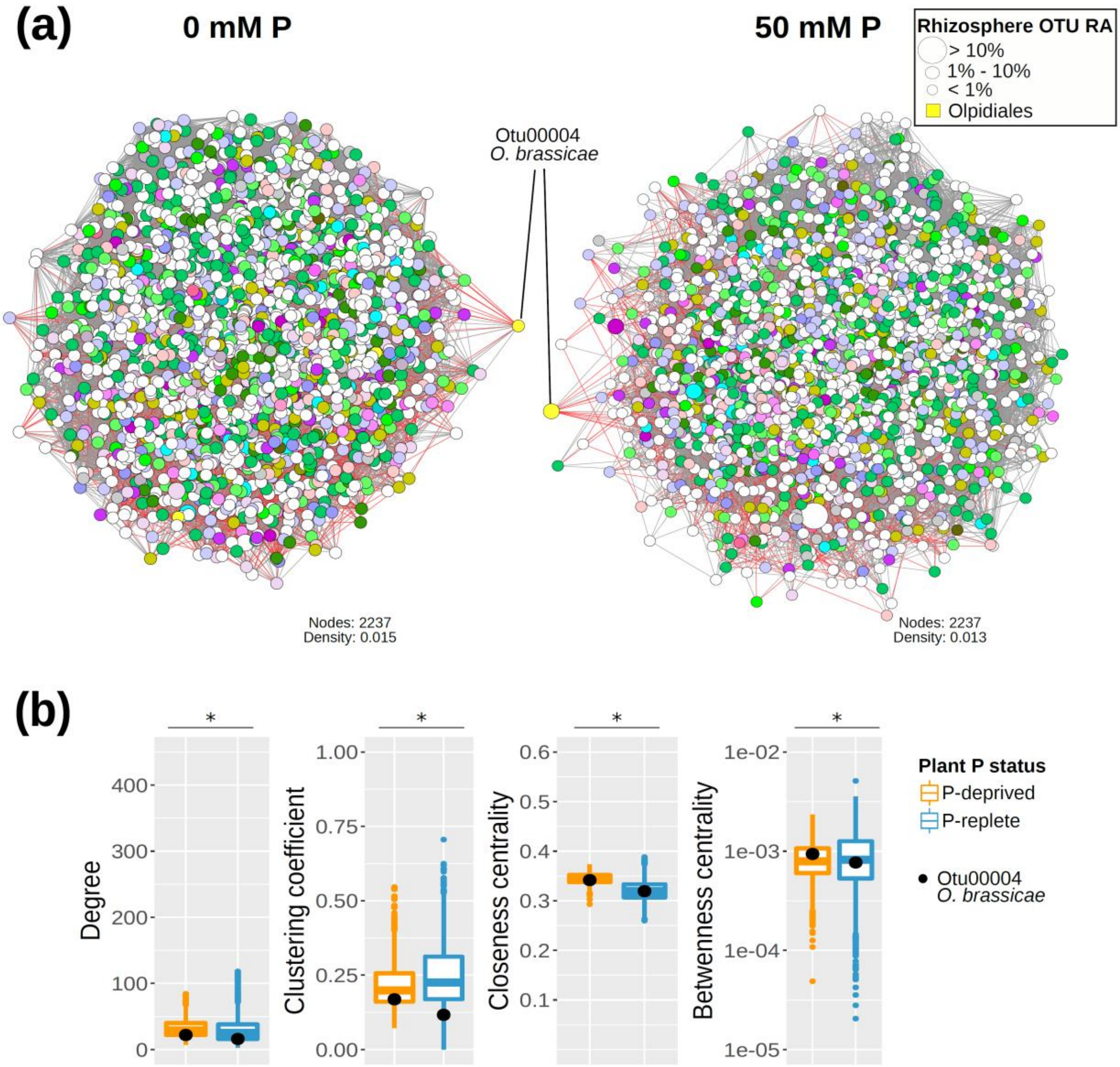

Fig. S9 Co-abundance networks of fungal OTUs in bulk soil under P-deprived (grown in 0 mM P) and P-replete conditions (50 mM P_K or P_Na). (a) Networks constructed based on the correlations between fungal OTUs abundances. Nodes represent OTUs and are colored by their order (as in Figs. 2, 3). The size of the node indicates the relative abundance (RA) of the OTU. Edges are colored accordingly to the Spearman correlation coefficient: red indicates negative correlation ($\rho < -0.6$) and grey indicates positive correlation ($\rho > 0.6$). **(b)** Differences between bulk soil fungal networks under P-deprived P (0 mM P) and P-replete conditions (50 mM P_K or P_Na). Asterisks indicate significant differences (Wilcoxon test $P < 10^{-4}$). Black dots indicate the values for Otu00004 *O. brassicae*. The whiskers of the box-plots depict the dispersion of the data (1.5 × interquartile range).

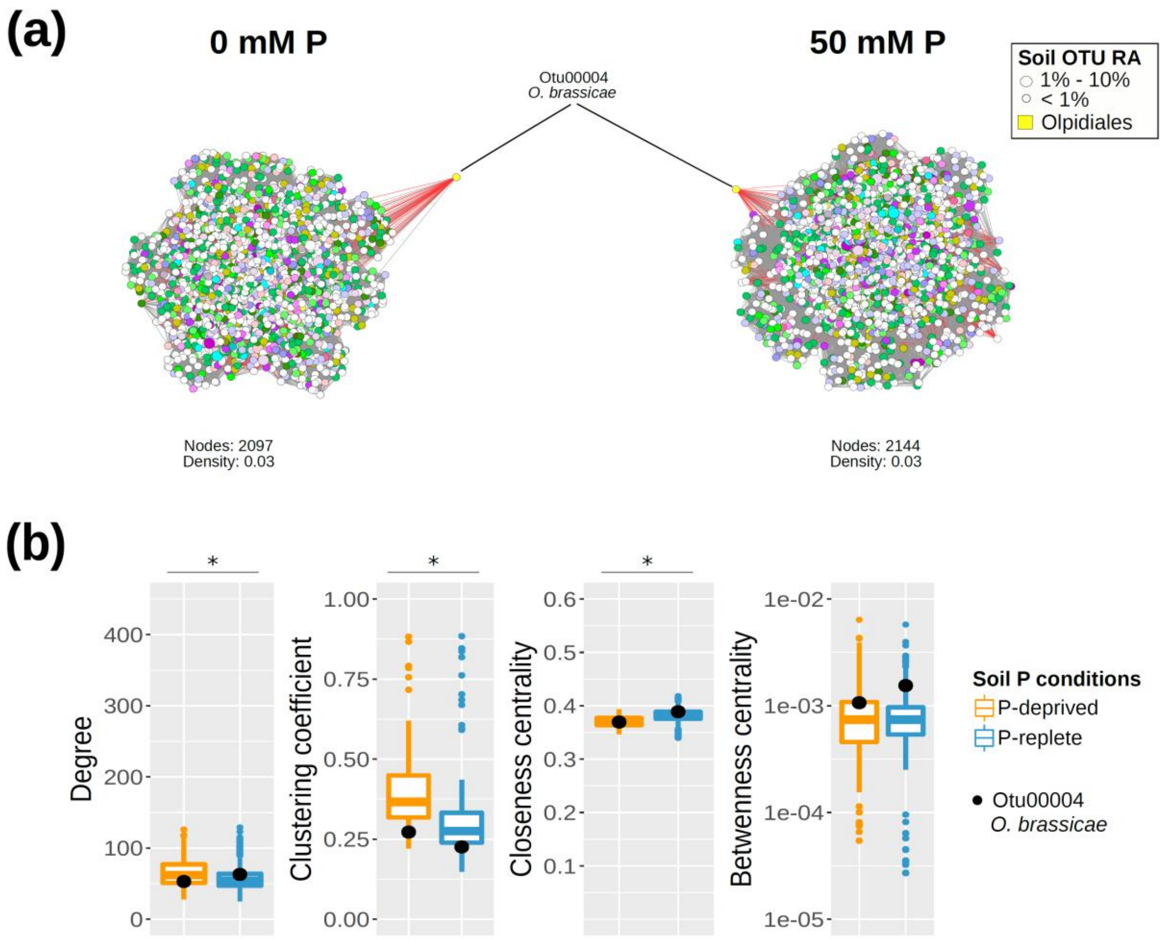

Fig. S10 Physiological responses of WT and mutant plants grown in soil under P-deprived and P-replete conditions. *A. thaliana* Col-0, *pho2*, *phr1* and *phr1 phl1* plants were pre-grown in sterile conditions and transferred to low P soil with ('50 mM P_K') or without P amendment ('0 mM P_water'). Shoot fresh weight (FW), shoot P concentration and shoot *AT4* transcript levels were measured. The experiment was performed twice with $n = 4 - 6$ samples composed of three pooled plants. Means and standard errors are shown, a-c indicate significant differences between the treatments (ANOVA followed by Tukey's test, $P < 0.05$). "n.a." indicates that no samples could be collected for *phr1 phl1* plants in 0 mM P soil.

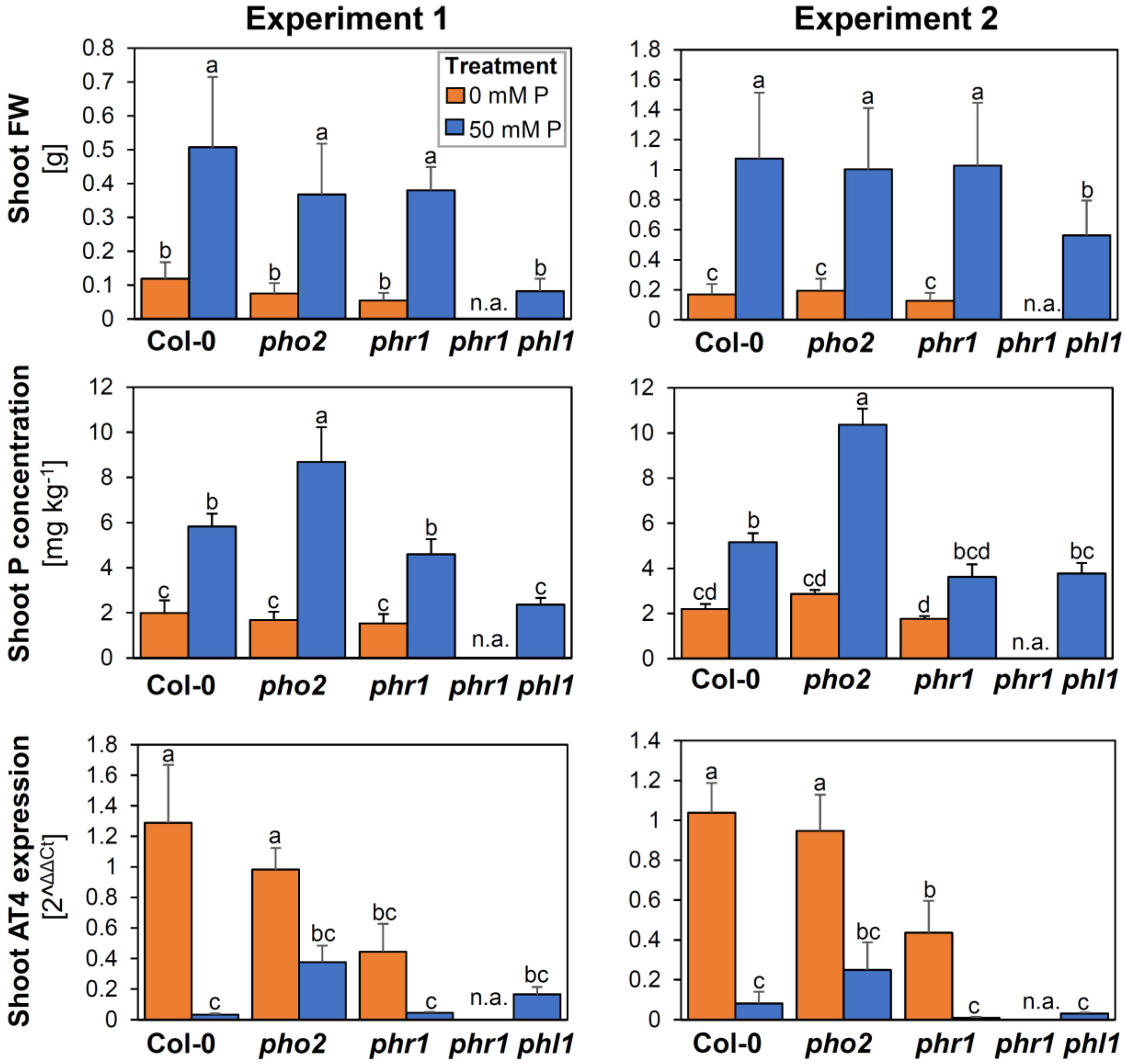

Fig. S11 Root-associated fungal communities in *A. thaliana* wild type (Col-0) and mutant plants. Col-0, *pho2*, *phr1* and *phr1 phl1* plants were pre-grown in sterile conditions and transferred to low P soil with ('50 mM P_K') or without P amendment ('0 mM P_water'). **(a)** Fungal alpha-diversity estimated by Shannon's diversity index. a-c indicate significant differences between treatments within compartments and experiments (ANOVA followed by Tukey's test, $P < 0.05$). The whiskers of the box-plots depict the dispersion of the data (1.5 × interquartile range). **(b)** Constrained analysis of principal coordinates (CAP) using 'plant-line' as grouping factor. The *P*-value indicates the significance of the effect of the plant-line on sample separation. **(c)** Relative abundance of main fungal orders in root, rhizosphere and bulk soil. The experiment was performed twice with n = 5-6 samples composed of three pooled plants.

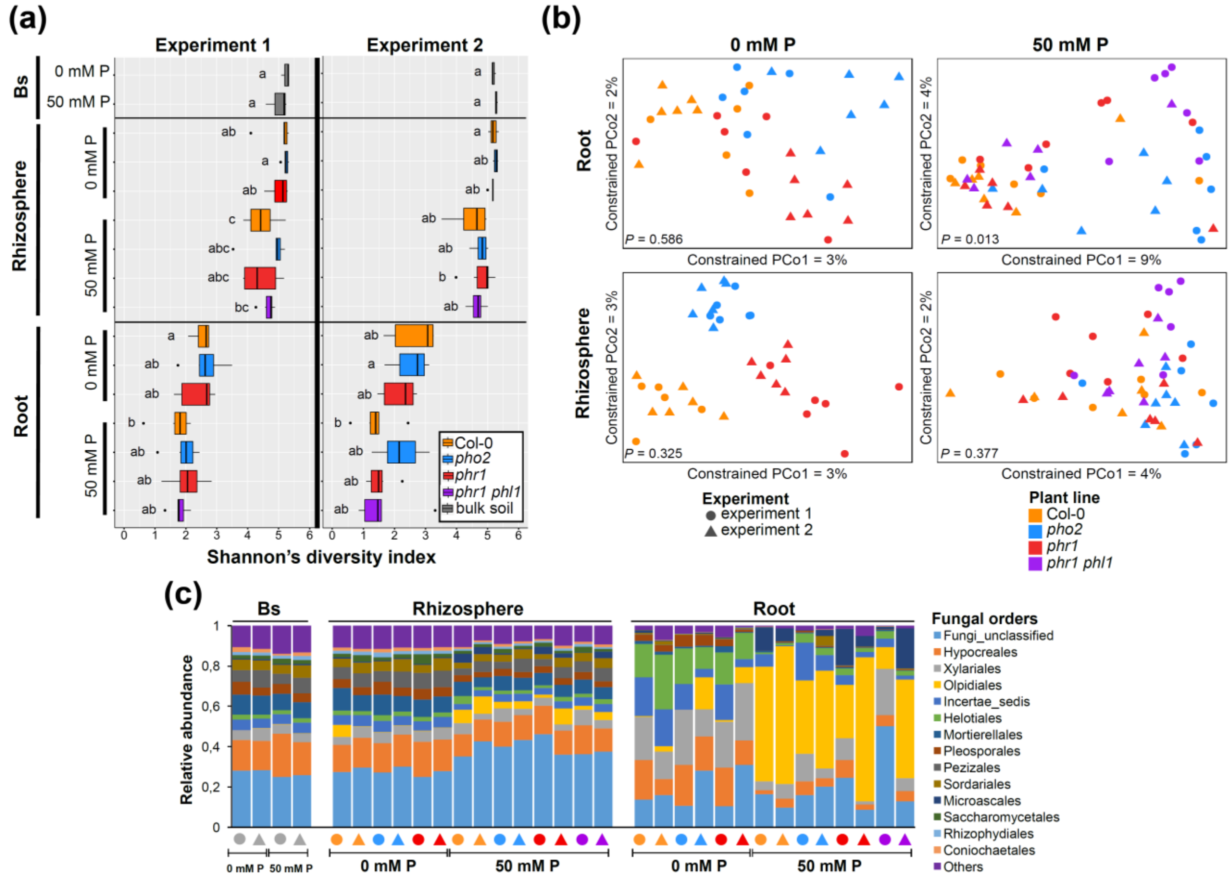

Fig. S12 *PHO2* gene expression in roots. *A. thaliana* Col-0, *pho2*, *phr1* and *phr1 phl1* plants were pre-grown in sterile conditions and transferred to low P soil with ('50 mM P_K') or without P amendment ('0 mM P_water'). The experiment was performed in parallel with the experiment for fungal community analysis (see Figs. 5 and S11), with n = 4 samples composed of three pooled plants. "n.a." indicates that no samples could be collected for *phr1 phl1* plants in 0 mM P soil. The whiskers of the box-plots depict the dispersion of the data (1.5 × interquartile range).

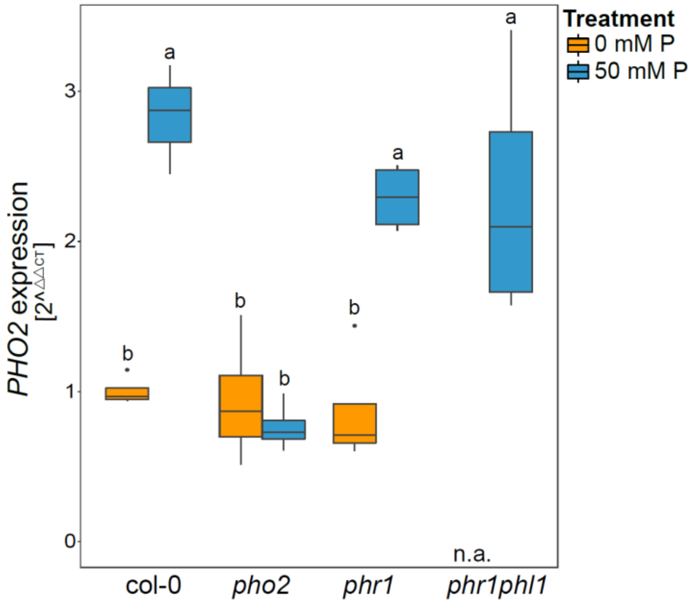

Fig. S13 Relative abundance (RA) of OTUs contributing to differences between plant lines under high-P conditions ('50 mM P_K') based on SIMPER analysis. The OTUs showing a significantly different RA between *A. thaliana* plant lines in root samples are indicated with an asterisk (Wilcoxon $P < 0.05$). Only OTUs that could be annotated at the order level are presented. The experiment was performed twice with n = 5-6 samples composed of three pooled plants. The whiskers of the box-plots depict the dispersion of the data (1.5 × interquartile range).

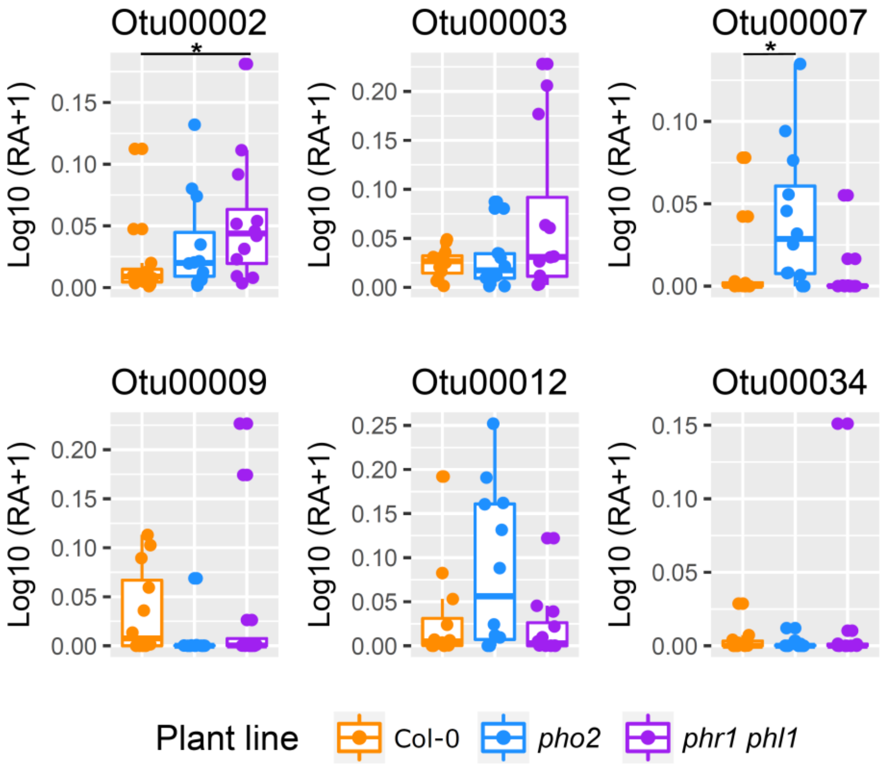

Table S1 Physico-chemical characteristics of the soil used.

Soil characteristics	Soil texture	Sandy-loam
	Humus	4%
	Clay	11%
	Slit	21%
	pH	6.6
Available [mg/kg] [a]	Nitrate	30.4
	Phosphorus	0.6
	Potassium	14
	Calcium	88.2
	Magnesium	19.3
Reserve [mg/kg] [b]	Phosphorus	16.8
	Potassium	139.3
	Calcium	2980
	Magnessium	330.1
	Br	0.7
	Mn	519
	Cu	11.9
	Fe	198

[a] determined by water extraction

[b] determined by ammonium-acetate-EDTA (AAE). Indicates plant available concentration.

Table S2 Solutions used for soil amendment.

	Soil amendment treatments								
Treatment name	0 mM P_water	0 mM P_K	0mM P_Na	1 mM P_K	1mM P_Na	20mM P_K	20mM P_Na	50 mM P_K	50mM P_Na
Added solution [a]	water	1.66 mM KCl	1.66 mM NaCl	potassium phosphate buffer	sodium phosphate buffer	potassium phosphate buffer	sodium phosphate buffer	potassium phosphate buffer	sodium phosphate buffer
Cation K or Na (mM) [b]	0	1.66	1.66	1.66	1.66	33.2	33.2	83	83
Added P / kg soil [c]	0	0	0	2.5	2.5	12.4	12.4	31	31

[a] Solutions used to amend the soil. 200 ml of the solution were added to 1 kg of soil. To maintain the soil pH at a stable level the solutions were adjusted to pH 7.5

[b] Two P sources were used to account for the cation added. Cation concentrations in the solutions used for soil amendment are indicated.

[c] Calculated as the amount of P [mg] per 1 kg of soil added in the amendment solutions

Table S3 Tagged primers used to prepare the ITS2 amplicon libraries.

Tag code[a]	Tagged fITS9 primers[b]	Tagged ITS4 primers[c]
0	catgcgGAACGCAGCRAAIIGYGA	catatgTCCTCCGCTTATTGATATGC
1	gcagtGAACGCAGCRAAIIGYGA	actgcTCCTCCGCTTATTGATATGC
2	tagctGAACGCAGCRAAIIGYGA	agctaTCCTCCGCTTATTGATATGC
3	gactgtGAACGCAGCRAAIIGYGA	acagtcTCCTCCGCTTATTGATATGC
4	cgtcgaGAACGCAGCRAAIIGYGA	tcgacgTCCTCCGCTTATTGATATGC
5	gtcgcGAACGCAGCRAAIIGYGA	gcgacTCCTCCGCTTATTGATATGC
6	acgtaGAACGCAGCRAAIIGYGA	tacgtTCCTCCGCTTATTGATATGC
7	cactacGAACGCAGCRAAIIGYGA	gtagtgTCCTCCGCTTATTGATATGC
8	tgacGAACGCAGCRAAIIGYGA	gtcaTCCTCCGCTTATTGATATGC
9	agtaGAACGCAGCRAAIIGYGA	tactTCCTCCGCTTATTGATATGC
10	atgaGAACGCAGCRAAIIGYGA	tcatTCCTCCGCTTATTGATATGC
11	tgcaGAACGCAGCRAAIIGYGA	tgcaTCCTCCGCTTATTGATATGC

[a] Tag codes and sequences as presented elsewhere (Gloor et al., 2010).

[b, c] Barcode sequences are presented in lowercase and the primer sequences in uppercase. Primer sequences are in 5’→ 3’orientation

Table S4 Sequence analysis summary.

Sample type	Total reads [a]		Fungal reads [b]		Fungal OTUs [c]	
	reads	St. dev.	reads	St. dev.	OTU number	St. dev.
bulk soil	24984.15	12416.2	24842.5	12350.1	1028.2	138.4
rhizosphere	24101.3	11430.9	23800.9	11239.1	948.5	160.4
root	21230.9	10913.1	11293.4	6951.8	148.3	64.3

[a] Number of reads per sample after chimera and low abundant OTU removal.

[b] Final number of fungal reads per samples after removal of non-fungal reads.

[c] Fungal OTU number per sample after removal of non-fungal reads.

Table S5 Primers used for RT qPCR.

Gene	AGI [a]	Forward primer [b]	Reverse primer [c]	Reference
UBQ10	At5g53300	CACACTCCACTTGGTCTTGCGT	TGGTCTTTCCGGTGAGAGTCTTCA	Balcerowicz et al., 2014
AT4	At5g03545	AATATGGCCATCCCCTAAAGAAAC	TAAACCGGAAACAAAGTAAACACG	Nilsson et al.,2007
PHT1;8	At1g20860	ACTGCAGAAAACGTCTACGACG	CAGCGATGATGGCTCCTAATTC	Nilsson et al., 2007
PHO2	At2g33770	GTGAAGGACCATTTTACGCACC	CCATATAAGCCTTGCACGCAG	Nilsson et al., 2007

[a] *Arabidopsis* gene identifier

[b, c] primer sequences are in 5'→ 3'orientation

Table S6 Effect of different factors on the alpha-diversity (ANOVA on Shannon's H index) and structure (PerMANOVA on Bray-Curtis dissimilarities) of fungal communities in the soil P amendment experiment. Samples from '0 mM P_water' treatment were excluded from this analysis. All the factors and their interaction were considered. Bold numbers indicate significant *P*-values.

Factor	Alpha-diversity		Community structure	
	R^2	*P*	R^2	*P*
**Global**				
Compartment	0.881	**2×10^{-16}**	0.418	**1×10^{-4}**
P source	0.004	**0.024**		0.122
P concentration		0.094	0.067	**1×10^{-4}**
Compartment x P source		0.528		0.733
Compartment x P concentration	0.023	**7×10^{-5}**	0.042	**0.034**
P source x P concentration	0.006	**6×10^{-4}**		0.131
Compartment x P source x P concentration		0.184		0.774
**Root**				
P source		0.296		0.387
P concentration	0.159	**0.022**	0.185	**1×10^{-4}**
P source x P concentration	0.228	**0.004**		0.27
**Rhizosphere**				
P source	0.072	**0.023**		0.057
P concentration	0.348	**1×10^{-4}**	0.204	**1×10^{-4}**
P source x P concentration		0.186		0.274
**Bulk soil**				
P source		0.284		0.963

P concentration	0.932	0.793
P source x P concentration	0.119	0.145

Table S7 Effect of different factors on the alpha-diversity (ANOVA on Shannon's H index) and structure (PerMANOVA on Bray-Curtis dissimilarities) of fungal communities in the soil P amendment experiment (the plant P-status was included as a factor; See Figs 1, S4, S5). Samples from '0 mM P_water' treatment were excluded from this analysis. All the factors and their interaction were considered. The 'P concentration' was considered as a factor nested within the 'P status' (P status / P concentration). Bold numbers indicate significant *P*-values.

Factor	Alpha-diversity		Community structure	
	R^2	*P*	R^2	*P*
**Global**				
Compartment	0.881	**2×10^{-15}**	0.418	**1×10^{-4}**
P source	0.004	**0.024**		0.124
P status		0.116	0.049	**1×10^{-4}**
Compartment x P source		0.551		0.735
Compartment x P status	0.017	**2×10^{-4}**	0.027	**0.003**
P source x P status	0.005	**0.011**		0.223
P status / P concentration		0.107	0.018	**0.029**
Compartment x P source x P status		0.382		0.722
Compartment x P status / P concentration		0.084		0.608
P source x P status / P concentration	0.008	**0.004**		0.148
Compartment x P source x P status / P concentration		0.151		0.678
**Root**				
P source		0.296		0.393
P status		0.089	0.127	**1×10^{-4}**

P source x P status	0.064	**0.045**		0.388
P status / P concentration	0.125	**0.022**	0.060	**0.042**
P source x P status / P concentration	0.153	**0.010**		0.240
**Rhizosphere**				
P source	0.072	**0.0231**		0.058
P status	0.343	**7×10^{-6}**	0.161	**1×10^{-4}**
P source x P status		0.139		0.344
P status / P concentration		0.873		0.175
P source x P status / P concentration		0.254		0.248
**Bulk soil**				
P source		0.284		0.966
P status		0.607		0.608
P source x P status		0.289		0.269
P status / P concentration		0.925		0.774
P source x P status / P concentration		0.090		0.173

Table S9 Effect of different factors on the alpha-diversity (ANOVA on Shannon's H index) and structure (PerMANOVA on Bray-Curtis dissimilarities) of fungal communities in the *A. thaliana* mutants experiment (See Figs 5, S11). All the factors and their interaction were considered. Bold numbers indicate significant *P*-values.

Factor	Alpha-diversity		Community structure	
	R^2	*P*	R^2	*P*
**Global**				
Compartment	0.853	**2×10^{-16}**	0.321	**1×10^{-4}**
Treatment	0.041	**3×10^{-13}**	0.099	**1×10^{-4}**
Genotype		0.065	0.017	**0.0147**

Experiment		0.852	0.024	**1×10^{-4}**
Compartment x Treatment		0.201	0.038	**1×10^{-4}**
Compartment x Genotype		0.830		0.429
Treatment x Genotype		0.122	0.015	**0.005**
Compartment x Experiment		0.135	0.013	**6×10^{-4}**
Treatment x Experiment		0.707	0.007	**0.027**
Genotype x Experiment		0.925		0.098
Compartment x Treatment x Genotype		0.556		0.098
Compartment x Treatment x Experiment		0.671		0.128
Compartment x Genotype x Experiment		0.380		0.257
Treatment x Genotype x Experiment		0.572		0.105
Compartment x Treatment x Genotype x Experiment		0.477		0.610
**Root**				
Treatment	0.274	**4×10^{-7}**	0.206	**1×10^{-4}**
Genotype		0.275		0.053
Experiment		0.316	0.054	**1×10^{-4}**
Treatment x Genotype		0.178	0.040	**0.009**
Treatment x Experiment		0.633		0.051
Genotype x Experiment		0.584		0.125
Treatment x Genotype x Experiment		0.474		0.293
**Rhizosphere**				
Treatment	0.325	**4×10^{-8}**	0.188	**1×10^{-4}**
Genotype		0.219		0.251
Experiment		0.233	0.059	**1×10^{-4}**
Treatment x Genotype		0.664		0.234

Treatment x Experiment		0.964		0.057
Genotype x Experiment		0.710		0.291
Treatment x Genotype x Experiment		0.663		0.178
**Bulk soil**				
Treatment		0.518		0.205
Experiment		0.357	0.153	**4×10^{-4}**
Treatment x Experiment		0.194		0.407

Table S10 Within-treatment analysis of the effect of different factors on the alpha-diversity (ANOVA on Shannon's H index) and structure (PerMANOVA on Bray-Curtis dissimilarities) of fungal communities in the *A. thaliana* mutants experiment (See Figs 5, S11). Samples were separated according to the treatment and compartment; all the factors and their interaction were considered. Bold numbers indicate significant *P*-values.

Factor	Alpha-diversity		Community structure	
	R^2	*P*	R^2	*P*
**0 mM P root**				
Genotype		0.310		0.477
Experiment		0.766	0.075	**8×10^{-4}**
Genotype x Experiment		0.755		0.366
**0 mM P rhizosphere**				
Genotype		0.231		0.131
Experiment		0.238	0.107	**1×10^{-4}**
Genotype x Experiment		0.855		0.0514
**50 mM P root**				
Genotype		0.185	0.133	**0.003**

Experiment	0.289	0.102	**3×10^{-4}**
Genotype x Experiment	0.419		0.121
**50 mM P rhizosphere**			
Genotype	0.425		0.241
Experiment	0.441	0.089	**1×10^{-4}**
Genotype x Experiment	0.700		0.260
